# Health system readiness for immediate Kangaroo Mother Care (iKMC) among network of care facilities in the Sidama region, Ethiopia: a mixed-method study

**DOI:** 10.12688/gatesopenres.16395.1

**Published:** 2026-09-01

**Authors:** Abiy Seifu Estifanos, Hailemariam Negash, Setisemhal Getachew, Dorka Woldesenbet Keraga, Akalewold Alemayehu, Mekdes Shifeta Argaw, Meles Solomon, Fitsum Woldegabriel Belay, Siren Rettedal, Sachiyo Yoshida, Anayda Portela

**Affiliations:** 1 Addis Ababa University, Addis Ababa, Addis Ababa, Ethiopia; 2 Addis Ababa University School of Public Health, Addis Ababa, Addis Ababa, Ethiopia; 3 Hawassa University College of Social Science and Humanities, Hawassa, Sidama Region, Ethiopia; 4 Hawassa University College of Medicine and Health Sciences, Hawassa, Sidama Region, Ethiopia; 5 Hawassa University College of Medicine and Health Sciences, Hawassa, Sidama Region, Ethiopia; 6 Ministry of Health of Ethiopia, Addis Ababa, Ethiopia; 7 University of Stavanger, Stavanger, Rogaland, Norway; 8 Stavanger University Hospital, Stavanger, Rogaland, Norway; 9 World Health Organization, Geneva, Switzerland

**Keywords:** immediate Kangaroo Mother Care, small and sick newborn care, health system, Ethiopia

## Abstract

**Introduction:**

Despite Ethiopia’s remarkable progress in reducing deaths among children under five, neonatal mortality remains high. The provision of immediate Kangaroo Mother Care (iKMC) for clinically unstable preterm and low-birth-weight (LBW) newborns has proven effective in lowering neonatal mortality related to preterm newborns. This study aimed to assess Ethiopia’s health system readiness to integrate iKMC into Level 2 small and sick newborn care (SSNC) units, with a focus on a selected network-of-care facilities in the Sidama region.

**Methods:**

We employed a mixed-method design. Through a cross-sectional quantitative facility mapping across a network of 25 facilities, data were electronically captured and analyzed in SPSS using descriptive statistics. The qualitative assessment included in-depth interviews and focus group discussions with mothers and family members of preterm or LBW newborns and a range of stakeholders. Data were coded, thematized, and analyzed using the World Health Organization health systems building blocks and the SSNC continuum of care.

**Results:**

Out of the 25 facilities, 8 (32.0%) had inpatient newborn care units (NCUs) and attended to 16,843 (68.4%) of all mapped births, 1,044 (96.6%) of all preterm births, and 1,143 (94.0%) of all LBW births recorded in the 12-months preceding the assessment. Four facilities with NCUs, admitting 93.4% of preterm and 88.2% of LBW newborns from the catchment area, were identified as eligible to introduce iKMC. All four iKMC-eligible facilities provided antenatal corticosteroids, had trained dedicated health workers to care for preterm or LBW newborns, and offered KMC. Although insufficient, they also had SSNC devices and infrastructure. The qualitative assessment identified facilitators and barriers related to the health system, families’ knowledge, attitudes, engagement, and support, and mothers’ household responsibilities.

**Conclusion:**

Despite notable health system gaps, hospitals with inpatient NCUs demonstrated modest readiness and willingness to introduce iKMC and create a mother-newborn couplet model of care approach.

## Introduction

In 2022, an estimated 107,000 neonates died in Ethiopia, accounting for 60% of all under-five deaths in the country ^1^ which puts Ethiopia among the five countries with the highest burden of neonatal mortality worldwide. Nearly half of neonatal deaths worldwide occur on the first day of life and three-quarters within the first week. ^2^ In 2020, Ethiopia was estimated to have approximately 495,900 preterm births annually, with a preterm birth rate of 12.9%, the fifth highest burden globally. ^3^ While the exact burden of low birth weight (LBW) in Ethiopia remains unknown, ^4^ using the regional LBW rate of 16% for Ethiopia, ^5^ the estimated LBW burden in 2020 would be 603,537.

Prematurity related complications, intrapartum related events, and infections are the three leading causes of neonatal deaths, accounting for 74% of neonatal deaths globally ^6^ and approximately 90% in Ethiopia. Data from five large hospitals in Ethiopia indicate that the top three causes of death among preterm newborns are respiratory distress syndrome (45%), neonatal infections (30%), and birth asphyxia (13%). ^7^ The majority of these deaths are considered preventable with the provision of good quality care. ^8^

Kangaroo Mother Care (KMC) is a proven intervention that significantly reduces mortality, infection, and hypothermia among preterm newborns. ^9^ A 2016 Cochrane review reported a 40% reduction in mortality among preterm newborns receiving KMC compared to those who are given conventional care in an incubator or under a radiant warmer. KMC was also associated with a 65% reduction in nosocomial infection/sepsis, and a 72% reduction in hypothermia. ^10^ Despite this strong evidence global coverage of KMC remains very low. ^11^

A multi-country implementation study in Ethiopia (4 sites) and India (3 sites) demonstrated that facility-initiated KMC is achievable and scalable within routine health systems through strong government leadership and ownership, health system capacity strengthening, and the empowerment of mothers and families. ^12^ In Ethiopia, the four study sites achieved a population-level effective KMC coverage ranging from 54% to 82% – defined as at least 8 hours of skin-to-skin contact plus exclusive breastfeeding in the 24 hours before discharge. ^13^ Another multi-country World Health Organization (WHO) coordinated randomized controlled trial on immediate KMC (iKMC), conducted around the same time, enrolled newborns weighing 1,000–1.799 grams and compared outcomes between two groups: those who received iKMC within two hours after birth, irrespective of clinical stability, and those who received KMC after they were stabilized. The iKMC group had 25% lower mortality at 28 days of life (relative risk of death, 0.75; 95% confidence interval [CI], 0.64 to 0.89; P = 0.001). ^14^

In light of this new evidence, the WHO updated its recommendations. Previously, KMC was recommended for newborns weighing less than 2,000 grams, after clinical stabilization. in hospital settings. The updated guidance recommends initiating KMC immediately after birth for all preterm or LBW newborns, without waiting for clinical stabilization, and providing it at both facility and community levels. The WHO also revised recommendations on the duration of skin-to-skin contact for 8–24 hours per day, along with exclusive breastfeeding. ^15^

Similarly, Ethiopia’s Ministry of Health has developed a national KMC technical and implementation guideline. Ethiopia’s 2023 KMC technical and implementation guideline, aligned with WHO’s revised recommendations, recommends providing KMC immediately after birth to both stable and unstable preterm newborns (<37 weeks of gestation) and to those with LBW (<2,500 grams). ^16^ The guideline emphasizes the need to reorganize neonatal units to accommodate mothers of unstable newborns within inpatient maternal neonatal intensive care units (M-NCUs) aiming for non-separation. Once these sick preterm or LBW newborns are stabilized, they are transferred to KMC wards designated for stable infants. For newborns who are stable at birth, the guideline recommends admission to the stable newborns’ KMC ward if they weigh less than 2,000 grams. It also recommends providing KMC in the postnatal ward for stable newborns weighing between 2,000 and < 2,500 grams during the 24–48 hours before discharge.

Successful implementation of iKMC requires redesigning health facility infrastructure and care environments as well as equipping maternal and newborn health workers with the skills and resources to support mothers or family members in providing care for newborns. This includes ensuring access to space, resources, and support in the operating theater (OT), labor and delivery (LD), and Small and Sick Newborn Care (SSNC) unit. However, there is limited experience on how to effectively scale up iKMC within routine health systems settings, particularly at Level 2 SSNC units in contexts such as Ethiopia. These units should be equipped to provide thermal care, feeding support, infection management, respiratory support, including a Continuous Positive Airway Pressure (CPAP) device, jaundice management, patient monitoring, and safe referral.

The WHO is coordinating a multi-country iKMC implementation research (iKMC IR) study in four countries: two in Africa (Ethiopia and Nigeria) and two in Southeast Asia (Bangladesh and India). The research is organized into two phases: the first phase to develop an implementation model for each country’s context, and a second phase to scale up and evaluate the optimized implementation model. This study is part of the first phase of the iKMC implementation research in Ethiopia and aimed to assess the readiness of Ethiopia’s health system for the successful integration of iKMC into Level 2 SSNC units, focusing on a selected network of facilities in the Sidama region.

## Methods

We employed a mixed-methods research design to assess the health system’s readiness to provide iKMC as an integrated component of care for preterm or LBW newborns. ^17^

### Study context

In Ethiopia, phase 1 was conducted at a study site with a network of 25 facilities (8 with inpatient newborn care units (NCUs) and 17 birthing facilities) serving an estimated catchment population of 588,714, and an expected annual birth rate of 10,548. This network of facilities serves Hawassa city and its surrounding areas in the Sidama region, one of Ethiopia’s 12 regions.

The study site was selected in consultation with Ethiopia’s Ministry of Health. Key criteria included: strong commitment and leadership of regional health bureaus to support the study; the presence of a research partner (Hawassa University) to collaborate with Addis Ababa University in implementing the study; the availability of basic childbirth care services and SSNC services which had been strengthened through the Saving Little Lives project ^18^; and reasonable proximity of the sites to Addis Ababa to facilitate access for collaborators from the WHO, the Ministry of Health, and Addis Ababa University.

In Ethiopia, literacy among women aged 15 to 49 stands at 47.6%, with notable disparities between urban and rural populations. In urban areas, 66.8% of women are literate, compared to just 38.6% in rural settings. In the Southern Nations, Nationalities, and Peoples’ Region (SNNPR), where the Sidama region was located prior to gaining regional statehood in 2020, the literacy rate was 42.2%. Consistent with the national estimate, 47.5% of women in the SNNPR gave birth in a health facility. The study site represents both urban and rural populations. In the region where these districts are located, most households fall in the middle-to-upper wealth tiers, with a smaller proportion in the highest quintile. ^19^

### Facility mapping

A cross-sectional facility assessment was conducted across a purposively selected network of 25 facilities (6 private, 1 non-governmental, and 18 public facilities) in the Sidama region, which was chosen as the site for the first phase of iKMC IR. The site was selected to represent a district-level referral linkage to the care of small and sick newborns with Level 2 SSNC units, and all facilities within the district that provide childbirth or SSNC were included. Data for this study were collected between December 11, 2023, and April 15, 2024. Facilities that provided inpatient newborn care and in which at least 80% of preterm or LBW newborns in the catchment were born in were considered eligible to introduce iKMC (4 out of the 25 facilities).

The objectives of the mapping were firstly to identify and locate all birthing facilities and facilities with an SSNC unit within the study areas. Secondly, to enumerate the following across the network of facilities: admissions of pregnant women for childbirth, total live births, preterm or LBW births, and inpatient NCU admissions of preterm or LBW newborns. Finally, to document additional details – including infrastructure, services, and related components in facilities designated as iKMC implementing facilities. This detailed assessment allow us to understand: where most births, particularly preterm or LBW births, are occurring; what percentage of eligible preterm or LBW infants in the study area are being cared for in iKMC implementing facilities; how many preterm or LBW infants admitted to SSNC units are inborn versus outborn; where to direct implementation efforts – whether in iKMC facilities, lower-level, or referral facilities; the volume and direction of referrals for pregnant women and preterm or LBW newborns; where to focus efforts to strengthen referral linkages; and the overall service readiness of iKMC implementing facilities, especially inpatient NCU, including identification of major gaps requiring immediate action. Service readiness at the four iKMC-eligible facilities was evaluated by observing the various elements of Level 2 SSNC units outlined above.

The WHO study team drafted a central harmonized facility-mapping tool (Supplementary material), which was reviewed and adapted by research teams across the four countries participating in the iKMC implementation research to reflect their specific contexts. The tool collected data on service delivery and facility readiness, including the number of pregnant women admitted, live births, preterm births, LBW births, and small and sick newborns admitted to the NCU. It also captured data on infrastructure, human resources, and other health system factors relevant to inpatient newborn care and iKMC implementation. Data collection forms were digitized using REDCap, an electronic data collection platform, and securely hosted on a password-protected server.

The assessment was conducted by four research assistants, each with a master’s degree in public health and experience in quantitative methods, recruited from the same region. The research assistants received training organized by the WHO on the assessment protocol, REDCap – electronic data collection platform, and data collection procedures. Additional training sessions were held on-site by the Ethiopia study team to further enhance the research assistants’ skills and confidence with the data collection tools and field protocols. The assessment involved visits to both facilities with inpatient newborn care services and birthing facilities, where the research assistants conducted facility observations, extracted data from the relevant registers, and administered tools to collect quantitative data from facility managers and heads of the delivery and NCUs.

Data quality was enhanced through on-site supportive supervision by the Ethiopia study team members and the review of the submitted data. Once collected, the data were exported to SPSS for descriptive analysis.

### Qualitative methods

***Design***

We applied a phenomenological cross-sectional qualitative study design. We planned to conduct a total of 75 in-depth interviews (IDIs) and 8 focus-group discussions (FGDs) ( Table 1). The final number of IDIs and FGDs conducted was decided by data saturation.

**Table 1.** Qualitative data collection sampling plan, Ethiopia.

Participant type	Planned
**In-depth interviews**	**75**
Mothers of preterm, LBW, or sick newborns	10
The newborn’s family members (companion of the mother of preterm or LBW newborns)	10
The newborn’s maternal or paternal grandparent	10
Childbirth healthcare workers	10
Childbirth care unit heads	5
SSNC unit heads	5
SSNC healthcare workers	10
*Decision maker (Health facility managers)*	
Policy makers	5
Stakeholders	5
MCH case team leads	5
**Focus group discussions**	**[8]**
Mothers who recently gave birth	4
Community stakeholders	4

***Study participants***

Participants of the IDIs included mothers and family members of preterm or LBW newborns, health workers and heads of the delivery and SSNC units, as well as decision makers at the facility, regional, and national levels. FGDs were held with mothers who had recently given birth in the selected facilities and community stakeholders. They were systematically and purposively recruited from five hospitals, birthing facilities, and their surrounding communities to gain rich data on Level 2 SSNC and readiness for iKMC integration. The study participants for the IDIs were systematically and purposefully selected with the aim of obtaining complete and rich data surrounding Level 2 SSNC and readiness for iKMC integration.

***Data collection procedures***

We developed FGD and IDI tools to collect qualitative data ( Table 2). The instruments included assessment of the acceptability of introducing iKMC. Since iKMC was not yet practiced and M-NCUs were not available in Ethiopia at the time of the study, the research assistants used a video from Malawi to introduce the concept to participants. ^20^ Participants were then asked about the facilitators and barriers to implementing iKMC in facilities or adopting it within families and communities.

**Table 2.** Summary of qualitative interview and discussion topic areas and key questions.

Topic/theme	IDI: Key questions	FGD: Key questions	Key probes
**Antenatal care**	• Can you tell me about your pregnancy experience? • Did you attend ANC? How many times? Why/why not? • Were you informed about the risks of a preterm/LBW/sick baby? • Did you receive advice on the place of birth or newborn care? • What support or information did you receive?	• Do most women attend ANC? When do they start and how often? • Is ANC considered important? Why/why not? • What barriers prevent women from seeking ANC? • Who influences ANC decisions?	• Number of visits • Timing of first visit • Family involvement (husband, in-laws) • Access barriers (cost, distance, transport) • Risk identification and counselling • Information on KMC, breastfeeding • Why/why not • Please explain
**Childbirth care**	• Where did you give birth and why? • Did risk status influence place of birth? • Who supported you during labor? • What was your experience of care? • Were there challenges reaching the facility?	• Where do most women give birth and why? • What challenges exist in reaching facilities? • Why do some women give birth at home? • Are women satisfied with childbirth care?	• Transport availability (Ambulance) • Costs, distance, terrain • Companion during labor and delivery Public vs private preference
**Care of the baby after birth**	• How did you feel about the baby being born preterm/LBW/with problems? • How did you feel about your baby’s condition? • What care did the baby receive after birth? • When did breastfeeding start? • Do you have any suggestions about how care at birth and immediately after birth could be improved for women who give birth to preterm, LBW, or sick babies? • Did you receive any training on care at the birth of a newborn in the last year?	• Are babies weighed after birth? When is breastfeeding initiated? • Did any of you have difficulties with breastfeeding? • Do mothers receive support? • Were you happy with the way your baby was cared for while you were at the facility?	• Skin-to-skin care • Breastfeeding initiation timing • Feeding challenges • Separation of mother and baby • Did you have any concerns? • Please tell me more … • Training: • If so, in what areas/topics? Are there any other specific areas where you feel you require additional training? What are these?
**Referral**	• Were you or your baby referred? Why? • How was the referral experience? • Did you travel together with the baby?	• How are preterm/LBW/sick babies referred? • Do families accept referrals? • What challenges do they face?	• Transport access • Costs • Family decision-making • Acceptance of referral • Skin-to-skin during transportation • Why/why not?
**NICU or SNCU care/in the postnatal ward**	• Can you describe your NICU/SNCU experience? • Were you able to stay with your baby? • How was communication with staff? • Were there cost concerns?	• What is the community perception of NICU care? • Is it acceptable for mothers to stay in the hospital? • What challenges do they face staying?	• Accommodation, food • Staff availability and skills • Communication quality • Family support • Cost burden
**KMC: skin-to-skin**	• When did you learn about skin-to-skin care? • Were you supported in practicing it? • What was your experience? • Are you familiar with Kangaroo Mother Care? • How is KMC implemented in this facility?	• Have you seen or heard of this practice? • Would women accept it? Why/why not? • What is the first thing that comes to mind when you see the picture? • Have any of you heard or seen this practice before?	• Duration and frequency • Family support • Timing of initiation • Length of time to do it, how to do it? • Barriers (medical, cultural) • Why/why not? • Lack of support • Lack of knowledge
**KMC: exclusive breastfeeding**	• Did you exclusively breastfeed? • Did you give other feeds? Why? • What challenges did you face? • What suggestions do you have for us to support mothers and families better?	• Do women exclusively breastfeed? • Do they give other substances? Why? • How soon after birth is the baby first put to your breast? • Are mothers given breastfeeding support or advice?	• Colostrum feeding • Supplementary feeding (water, formula) • Breastfeeding support • Early initiation feasibility • Did you feel comfortable with this? • Was your baby comfortable?
**iKMC**	• This is what we call iKMC (Showing videos of iKMC). What do you feel when you look at that? • Do you think that it would be good if they started the KMC immediately here? • Do you think the family will refuse?	• How acceptable is iKMC? • What support is needed for mothers to stay? • Do you think women in the community would approve or disapprove of this practice?	• Zero separation feasibility • Facility space, beds, food • Family support systems • Facility readiness • Why/why not? • What concerns would they have about this practice?
**Discharge and follow-up in the home after discharge and care of the newborn in the home**	• Were you prepared for discharge? • Did health workers help her/you/the family to understand how to take care of herself and the baby at home? • Did you receive follow-up care? • What challenges do you face at home?	• Do women feel ready to go home? • What support do they receive after discharge?	• Home visits by health workers • Knowledge of newborn care • Family support • Challenges at home • What did they tell you about?

Seven research assistants with experience in qualitative research were recruited, four from Hawassa and three from Addis Ababa. Study investigators trained the research assistants in informed consent, qualitative data collection techniques, study objectives, and study procedures. Before full data collection, the research assistants piloted the data collection tools and procedures with mothers of preterm or LBW newborns at Hawassa University Comprehensive Hospital and Adare General Hospital. Based on the pretest findings, the interview guides were revised by paraphrasing questions, adding probes, and adjusting their sequencing.

IDIs and FGDs were conducted in waiting areas, designated rooms or spaces within facilities, and offices, with auditory and, when feasible, visual privacy maintained to ensure confidentiality. Consent was taken prior to initiating the discussions. Most of the IDIs and FGDs were conducted in Amharic, Ethiopia’s official and most widely spoken languages, while some were conducted in the Sidama Afoo language. The IDIs ranged from half and to two hours, while the FGDs lasted between one and three hours.

Interviews were intentionally sequenced to allow the next group of interviewees to reflect on observations from previous interviews, keeping the mother-small and sick newborn dyads at the center. First, mothers of preterm or LBW newborns admitted to the NCUs in the study sites were selected, invited to participate in the study, and interviewed. Second, their family members (companions), including the newborn’s maternal and paternal grandparents, when available, were invited and interviewed. Grandparents were included because they influenced the decision to seek care for the newborn in contexts such as Ethiopia. ^21^^,^^22^ Third, health workers involved in providing childbirth care services in the maternity unit and in the NCUs were invited and interviewed. Fourth, the heads of the delivery and NCUs were invited and interviewed, followed by decision makers at the facility, regional, and national levels. Women who had given birth in the recent past and community members, leaders, and stakeholders from the catchment population of the selected health facilities were invited to participate in the FGDs.

To ensure the quality of the qualitative component, an iterative approach was used throughout the data collection. After each interview, research assistants submitted a summary note to the study investigators the same day, and most transcriptions were sent within 48 hours for IDIs and within 72 hours for FGDs. IDIs and FGDs were recorded. Investigators reviewed the field notes and transcriptions and provided quick feedback. Virtual or in-person debriefing sessions with research assistants were also held twice weekly. These sessions allowed the study team to identify and discuss important themes, emerging ideas, and gaps in the data collection process, such as identify participants who can provide rich data on their experiences of care, and to propose adjustments as needed.

### Data analysis

We applied framework analysis to analyze the qualitative data. The WHO team provided templates to capture a summary of each IDI and FGD conducted. HN summarized the IDIs and FGDs into the templates and entered the summary notes into an online matrix developed by WHO that allowed for a rapid overview of findings by key categories constructed according to WHO health system building blocks as columns and core components of the continuum of maternal and newborn care and iKMC services as rows (antenatal care [ANC], childbirth care, postnatal care [PNC], referral systems, NCU, KMC-skin-to-skin contact, KMC-exclusive breastfeeding, iKMC, discharge and follow-up care at home, influencers, and communication delivery channels). ASE, FWB, AA, and MSA reviewed the summary of the IDIs and FGDs, along with the populated online matrix. For each theme, facilitators and barriers were identified and summarized, and solutions were suggested, supported by illustrative quotes. The quotes were selected for their clarity and uniqueness, with efforts made to balance the voices across the different categories of study participants.

HN further extracted data from the rapid analysis matrix for more in-depth analysis and summarized the extracted text into narratives using the WHO building blocks and the continuum of care. ASE critically reviewed and revised the summary narratives and quotes, ensuring consistency with the original data in the matrix. All authors reviewed the draft results.

As the authors of this study are not native English speakers, we used Chat GPT4 to support language editing. Specifically, we used prompts such as ‘improve the grammar of this text’ and ‘improve the readability of this text’, and carefully reviewed all outputs to ensure the intended meanings were not lost. We did not use AI to edit direct quotes to maintain the interviewees’ original voice.

### Reflexivity

While efforts have been made to ensure robust qualitative data collection, analysis, and interpretation, we acknowledge certain limitations related to our beliefs, assumptions, and background. For instance, the interpretation of the qualitative data may have been influenced by biases stemming from limited familiarity with the local health system and socio-cultural context of the study area, particularly among research team members based in Addis Ababa. Although Sidama Afoo-speaking research assistants were recruited, the team’s overall language proficiency in Sidama Afoo may have limited its ability to fully capture linguistic nuances in the data. In addition, iKMC was introduced in three of the four eligible facilities during the analysis and manuscript preparation. The involvement of co-authors in implementing iKMC may have influenced how the formative assessment data collected before iKMC was introduced at the study sites were interpreted.

The research assistants made significant efforts to build trust and establish rapport with the study participants by greeting and introducing themselves, providing an extended description of the study aims, ensuring that participation was completely voluntary, and assuring participants that their involvement in the study would not influence the care they were receiving or would receive. Although some participants might have felt comfortable speaking with unfamiliar individuals, we acknowledge that some responses may have been influenced by their perceptions of interviewers as outsiders. Despite the gender-balanced composition of the study team, the predominantly patriarchal context may have posed challenges for male research team members in fully understanding the lived experiences and emotions of mothers and female healthcare workers involved in the provision and receipt of SSNC.

### Ethics approval

Ethical clearance was obtained from the Institutional Review Board of the College of Health Sciences at Addis Ababa University (060/23/SPH) and from the WHO Ethics Review Committee (Protocol ID: ERC.0003880). In addition, support letters were secured from the Sidama Regional Health Bureau and distributed to all participating facilities. Each respective institution granted permission to access facility-level data. For the facility assessment, individual consent was not required, as the study did not involve personal or patient-level data. Informed written consent was taken for the IDIs and FGDs. All notes and summaries anonymized the participants’ identities.

## Findings

### Facility mapping findings

***Maternal and newborn care services***

**Figure 1.**
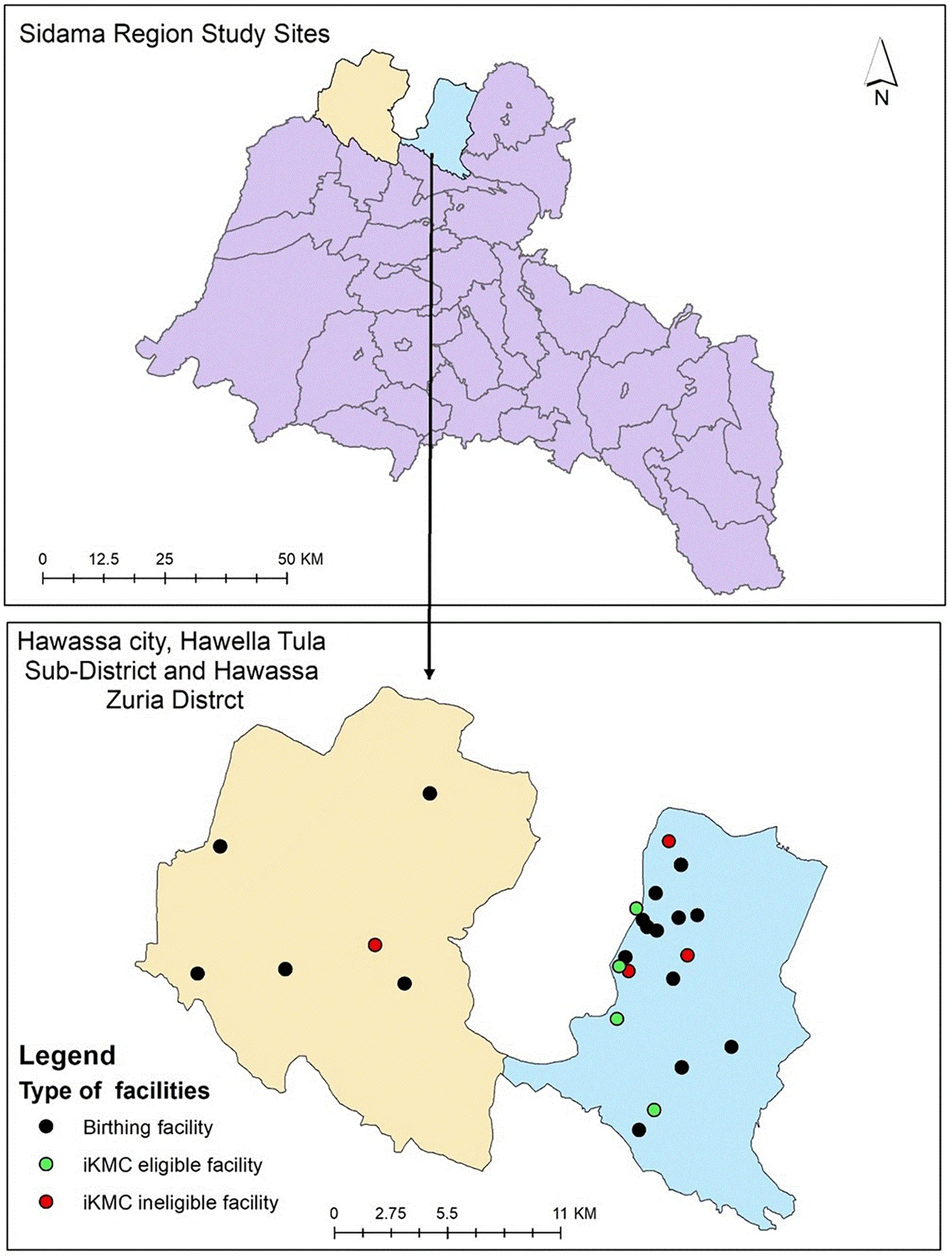
Map showing geographical distribution of health facilities assessed for iKMC-IR in Sidama region, Ethiopia, 2024.

Among the 25 health facilities assessed, 17 (68.0%) provided only childbirth care services (birthing facilities) and 8 (32.0%) had inpatient NCUs in addition to childbirth care services. 14 (82.4%) of the birthing facilities and half of (50.0%) the facilities with inpatient NCUs were public. Nearly all birthing facilities, 15 out of 17 (88.2%), provided Basic Emergency Obstetric and Newborn Care (BEmONC) services. ^23^ One birthing facility and all facilities with NCUs provided Comprehensive Emergency Obstetric and Newborn Care (CEmONC) services. Half (50.0%) of the facilities with NCUs were identified as eligible for iKMC implementation ( Table 3, Figure 1).

**Table 3.** Maternal and newborn care services profile of health facilities in Sidama region, Ethiopia, 2024.

Variables	Categories	Frequency	Percentage (%)
Maternal and newborn care services			
Facility type (n = 25)	Birthing facility	17	68.0
	Facility with inpatient NCU	8	32.0
Birthing facilities (n = 17)	BEmONC	15	88.2
	CEmONC	1	5.9
	Partial BEmONC *	1	5.9
Facilities with inpatient NCU (n = 8)	iKMC eligible	4	50.0
	iKMC ineligible	4	50.0
	BEmONC	0	0.00
	CEmONC	8	100.0
Ownership status			
Birthing facilities (n = 17)	Public	14	82.4
	Private	3	17.7
iKMC eligible (n = 4)	Public	3	75.0
	Private	1	25.0
iKMC ineligible (n = 4)	Public	1	25.0
	Private	3	75.0

* The birthing facility was designated as a BEmONC facility but lacked staff trained in BEmONC and therefore did not provide the full range of BEmONC functions at the time of assessment.

***Pregnant women’s admission for childbirth and the type of health facilities***

In the 12 months preceding data collection, there were 24,170 admissions of pregnant women for childbirth. Of these 16,539 (68.4%), occurred in CEmONC facilities with inpatient NCUs.

More than half of the childbirth admissions, totaling 13,386 (55.4%), occurred in iKMC eligible facilities, 7,631 (31.6%) in birthing facilities, and 3,157 (13.1%) in iKMC ineligible facilities.

**Figure 2.**
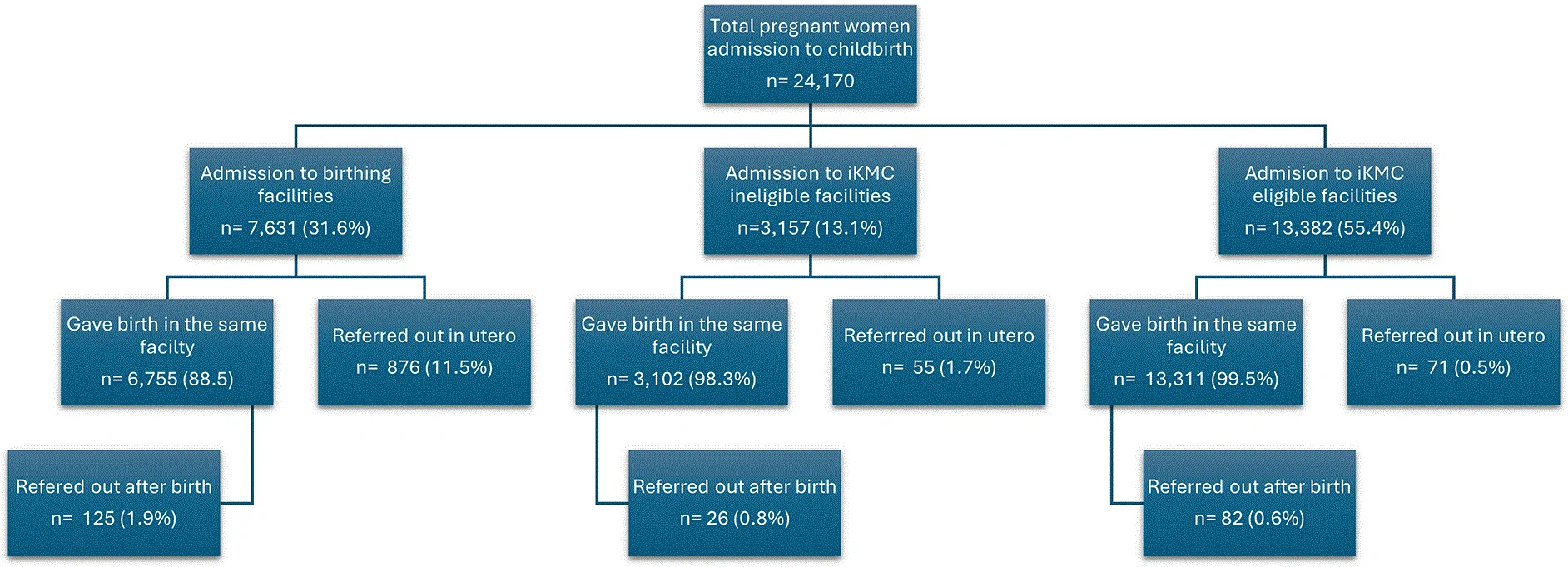
Flow diagram showing number of pregnant women admissions, inborn, in utero and after birth referral in iKMC-study sites, Sidama region, Ethiopia, 2024.

Regarding referrals, 876 (11.5%) of childbirth admissions in birthing facilities, 55 (1.7%) in iKMC-ineligible facilities, and 71 (0.5%) in iKMC-eligible facilities were referred out in utero to other facilities. In addition, among those who gave birth in the same facility where they were admitted, 125 (1.9%) from birthing facilities, 26 (0.8%) from iKMC-ineligible facilities, and 82 (0.6%) from KMC-eligible facilities were referred to other facilities after childbirth ( Figure 2).

Most of the in-utero referrals, 853 (85.1%) from BEmONC facilities and 126 (12.6%) from CEmONC facilities, were from those without inpatient NCUs. Additionally, 125 (53.7%) of post-birth referrals were from BEmONC facilities without inpatient NCUs ( Table 4).

**Table 4.** Birth admissions, births and in-utero and after birth referral cases of newborns by facility’s maternal and newborn care services in iKMC-IR study sites, Sidama region, Ethiopia, 2024.

Variables	Maternal and newborn care services, frequency (%)				Total
	Partial BEmONC facility	BEmONC facility	CEmONC facility with inpatient NCU		
			Yes	No	
Number of facilities	1 (4.0%)	15 (60.0%)	8 (32.0%)	1 (4.0%)	25 (100.0%)
Number of childbirth admission	428 (1.8%)	7,142 (29.6%)	16,539 (68.4%)	61 (0.3%)	24,170 (100.0%)
Number of in utero referrals	23 (2.3%)	853 (85.1%)	126 (12.6%)	0 (0.0%)	1,002 (100.0%)
Number of same facility births	403 (1.8%)	6,714 (29.2%)	15,788 (68.8%)	61 (0.3%)	22,966 (100.0%)
Number of preterm births *	0 (0.0%)	37 (3.4%)	1,044 (96.6%)	0 (0.0%)	1,081 (100.0%)
Number of low-birth-weight birth *	0 (0.0%)	69 (5.7%)	1,143 (94.0%)	4 (0.3%)	1,216 (100.0%)
Number of referrals after birth	0 (0.0%)	125 (53.7%)	108 (46.4%)	0 (0.0%)	233 (100.0%)

* Preterm and low birth weight data are not mutually exclusive.

***Service availability in iKMC eligible facilities***

Routine antenatal ultrasound, ANC services including protocols for identifying high-risk women, and antenatal breastfeeding counseling were available in all four iKMC-eligible facilities. However, antenatal KMC counseling was unavailable at three of the four facilities.

All iKMC-eligible facilities allowed a companion of choice during labor and birth, provided antenatal corticosteroids, had dedicated health workers to care for preterm or LBW newborns, had health workers trained in resuscitation for preterm or LBW newborns, and supported KMC for stable newborns. All facilities had resuscitation corners in both labor rooms and OTs. Additionally, all facilities were equipped with functional radiant warmers and preterm-sized bags and masks for newborns in the labor rooms and OTs.

Handwashing facilities with an uninterrupted water supply were available in the labor rooms and operating theatres (OTs) at only one of the four facilities. Functional digital weighing scales were present in the labor rooms of all facilities, in the OTs and NCUs of three facilities, and in the post-natal wards of only one facility ( Table 5).

**Table 5.** Maternal and newborn service availability in iKMC implementing facilities, Sidama region, Ethiopia, 2024.

Services	Governmental Referral Hospital	Governmental Primary Hospital	Non-governmental Maternal and Child Health Center	Governmental General Hospital
Is routine antenatal ultrasound available?	Yes	Yes	Yes	Yes
Antenatal care services	Yes	Yes	Yes	Yes
Is there protocol for identifying high risk women?	Yes	Yes	Yes	Yes
Is there antenatal breastfeeding counseling?	Yes	Yes	Yes	Yes
Is there antenatal of KMC counseling?	No	No	Yes	No
Childbirth services	Yes	Yes	Yes	Yes
Is companion of choice allowed?	Yes	Yes	Yes	Yes
Is antenatal corticosteroid given for preterm birth?	Yes	Yes	Yes	Yes
Are there dedicated healthcare workers to care for preterm or LBW at birth?	Yes	Yes	Yes	Yes
Is there a health worker trained in the resuscitation of a newborn born preterm or LBW?	Yes	Yes	Yes	Yes
Is there a hand washing facility with 24x7 water supply in the labor room?	No	No	Yes	No
Is functional digital weighing scale available in the labor room?	Yes	Yes	Yes	Yes
Is functional digital weighing scale available in the operation theatre?	Yes	No	Yes	Yes
Is functional digital weighing scale available in the NCU?	Yes	No	Yes	Yes
Is functional digital weighing scale available in the postnatal ward?	No	No	Yes	No
Is a newborn care corner available in the labor room and operating theatre?	Yes	Yes	Yes	Yes
Is a functional radiant warmer available in the labor room and operating theatre?	Yes	No	Yes	Yes
Are preterm size bag and mask available in the labor room and operating theatre?	Yes	Yes	Yes	Yes
Is KMC practiced for preterm or LBW newborns?	Yes	Yes	Yes	Yes

***Service readiness for SSNC***

iKMC-eligible facilities were equipped with thermal care devices, including room heaters, radiant warmers, and incubators. All facilities provided KMC services for preterm or LBW newborns. CPAP was also commonly used for respiratory support; however, two facilities used improvised CPAP with 100% oxygen. During oxygen therapy, newborns were monitored for oxygen saturation every 2 to 4 hours at three facilities. At one of the facilities, continuous monitoring is provided using a patient monitor. In terms of oxygen supply, only one of the four iKMC-eligible facilities was equipped with an oxygen plant that includes blenders. The other three facilities relied on oxygen cylinders, concentrators, and CPAP machines as their primary sources of oxygen. All facilities reported initiating breastfeeding within one hour of birth for stable newborns (born without complications) and offering assisted feeding as well as intravenous fluids and medication services for clinically unstable newborns.

In terms of infection prevention, detection, and management, all facilities had handwashing facilities within or at the entrance of their newborn units. However, three facilities experienced frequent water supply interruptions. Hand rubs and alcohol-based disinfectants were also used inside the units, though proper handwashing practices were not consistently observed in three of the facilities. One facility had full culture workout services and clinical protocols in place to prevent, detect, and manage of necrotizing enterocolitis, neonatal encephalopathy, and seizures.

Regarding referral pathways, all facilities communicated with the receiving facility through a liaison office. However, they reported a lack of communication when receiving newborns referred from lower-level facilities, and they were often transferred without being accompanied by a health worker. Most referred newborns arrived via public transportation with their family members.

The iKMC facilities had obstetricians, neonatal nurses, neonatologists/pediatricians, and midwives, and a significant proportion of the healthcare workers had received training in the management of preterm birth and in the provision of SSNC and KMC ( Tables 6 and 7).

**Table 6.** Health workforce data in iKMC-IR study site facilities, Sidama region, Ethiopia, 2024.

Maternal and neonatal health workforce		Governmental Referral Hospital			Governmental Primary Hospital			Non-governmental Maternal and Child Health Center			Governmental General Hospital		
		San	Ava	Vac	San	Ava	Vac	San	Ava	Vac	San	Ava	Vac
Maternal health workforce	Obstetricians	13	13	0	1	1	0	3	3	0	4	4	0
	GPs	2	2	0	0	0	0	3	3	0	7	7	0
	Nurses	0	0	0	0	0	0	7	7	0	4	4	0
	Midwives	97	94	4	24	21	3	13	13	0	62	49	13
Neonatal health workforce	Pediatricians	17	14	3	1	1	0	2	2	0	3	3	0
	GPs	0	0	0	2	2	0	3	3	0	3	3	0
	Nurses	15	15	0		13		5	5	0	16	16	0

San = sanctioned, Ava: available, Vac = vacant.

**Table 7.** Health workforce training profile at iKMC-IR study site facilities, Sidama region, Ethiopia, 2024.

Neonatal care trainings provided	Governmental Referral Hospital		Governmental Primary Hospital		Non-governmental Maternal and Child Health Center		Governmental General Hospital	
	Nurses trained	Doctors trained	Nurses trained	Doctors trained	Nurses trained	Doctors trained	Nurses trained	Doctors trained
Essential newborn care including neonatal resuscitation (all newborns)	14	0	7	0	5	0	8	0
Care at birth & neonatal resuscitation (Preterm births)	14	0	7	0	5	0	8	0
Thermal care & monitoring (Preterm births)	14	0	7	0	5	0	8	0
Breastfeeding and lactation support (Preterm births)	14	0	7	0	5	0	8	0
KMC	14	0	7	0	5	0	8	0
Respiratory support &CPAP	14	0	7	0	5	0	8	0
Management of common problems like jaundice & hypoglycemia	14	0	7	0	5	0	8	0

### Qualitative assessment findings

We conducted IDIs with a total of 63 participants: 15 with mothers of preterm, LBW or sick newborns; 10 with family members of preterm or LBW newborns (including 2 infant grandmothers/mothers-in-law); 12 with childbirth healthcare workers; 5 with childbirth care unit heads; 5 with NCU heads; 11 with NCU healthcare workers; and 5 with health facility managers. In addition, we conducted 8 FGDs: 4 in health facilities and 4 in the community. Half of the FGDs were held with women of all ages who had recently given birth to full-term newborns without complications or illness, mostly within the last three months. The remaining FGDs were conducted with community members, stakeholders, and leaders ( Table 8).

**Table 8.** Socio-demographic characteristics of the IDI and FGD participants.

Socio-demographic variables	Mothers of preterm or LBW babies	Family members of preterm or LBW babies			Healthcare workers				Health facility managers	Mothers who gave birth to full term babies	Community members, stakeholders, and leaders
					SSNB (NCU) HCWs		Childbirth workers				
		Fathers	Aunts/Great aunts	Grand mothers	General practitioners (GPs)	Nurses	Midwifes	Integrated Emergency Surgical Officers (IESO s)			
No. of participants	15	4	4	2	1	15	16	1	5	23	34
Age											
Min	16	24	23	35	-	25	24	-	30	18	23
Max	35	45	50	60	27	38	49	40	45	35	75
No. of living children											
Min	1	-	-	-	-	-	-	-	-	-	-
Max	4	-	-	-	-	-	-	-	-	-	-
Educational status											
Illiterate	0	0	0	2	-	0	0	-	-	3	3
Primary	7	0	0	0	-	0	0	-	0	7	8
High school	3	2	3	0	-	0	0	-	0	7	12
College or above	5	2	1	0	1	15	16	1	5	6	11
Religion											
Chirstian Orthodox	6	1	2	0	-		1	-	0	5	9
Christian Protestant	8	3	2	0	-	1	2	-	1	17	24
Muslim	1	0	0	2	-	0	0	-	0	1	1
Not recorded					1	14	13	1	4	0	0
Ethnicity											
Sidama	8	2	2	2	-	1	2	-	1	19	34
Oromo	1	1	0	0	-	0	0	-	0	2	0
Guraghe	0	0	0	0	-	0	0	-	0	1	0
Amhara	0	0	0	0	-	0	1	-	0	1	0
Not recorded	6	1	2	0	1	14	13	1	4	0	0
Marital status											
Currently married	15	4	4	2	-	1	2	-	1	23	32
Currently unmarried	0	0	0	0	-	-	1	-	0	0	2
Not recorded	-	-	-	-	1	14	13	1	4	0	0

**Figure 3.**
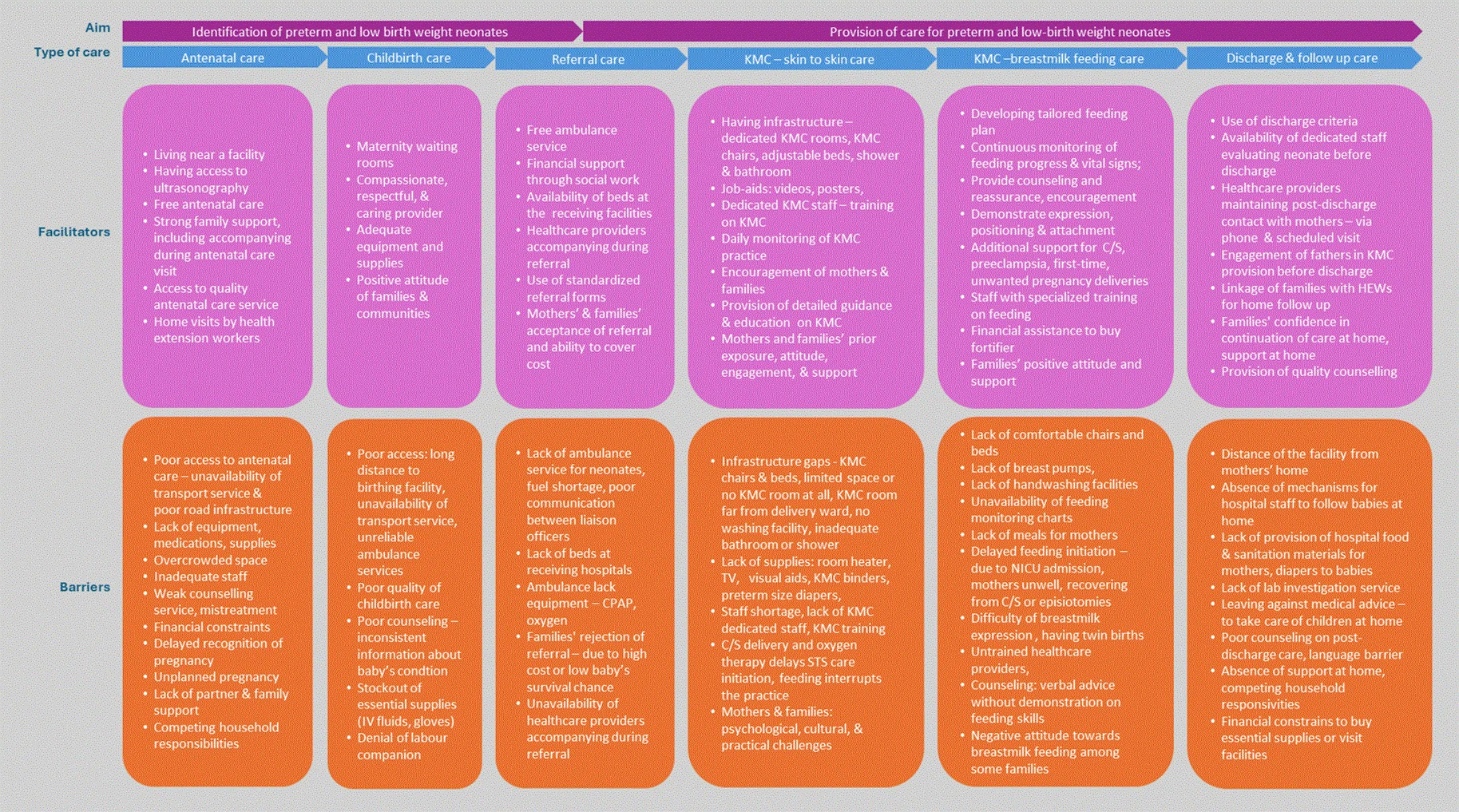
Facilitators and barriers to identification and provision of care for preterm and low-birth weight newborns, Sidama, Ethiopia.

We present the findings from the qualitative data analysis in three parts. First, we present participants’ perceptions and beliefs regarding preterm births. Second, we share the facilitators and barriers to identifying and providing care for preterm and LBW newborns—spanning ANC, childbirth, referral, KMC (skin-to-skin contact and breastmilk feeding), discharge, and follow-up care ( Figure 3). Finally, we highlight the facilitators and barriers to introducing iKMC within existing Level 2 SSNC units and to redesigning these units into M-NCUs.

***Perceptions and beliefs around preterm births***

Our study revealed that there was a misunderstanding regarding the causes of preterm birth. Some participants associated preterm birth with spiritual or moral transgressions, viewing it as a curse or punishment from God on parents, for misbehaving. Others believed in resulted from the mother’s or family’s sins, or from the mother having a sexual relationship before marriage.

“I feel that it is the consequence of what I did [my sin]. I had never experienced this, and it makes me sad [Crying]” (IDI – mother of preterm or LBW newborn).

“There are different beliefs from the community members culturally, the first thing is that she gave birth to a preterm baby because she was married before the age of her marriage [had premarital sex]” (FGD – community members, leaders and actors).

Mothers and family members reported feeling extremely scared and ashamed upon realizing that their newborn was born preterm or LBW. Many described fear at the sight of the newborn and persistent worry that the preterm newborn might stop breastfeeding.

“In the beginning, I was very scared … I haven’t seen children born preterm before, I was afraid when I saw them …” (IDI – mother of preterm or LBW newborn).

These feelings of fear, anxiety, stress, and lack of joy were often linked to the belief that such newborns were unlikely to survive or would not live long even if they do. Support from family members, including fathers, was often minimal, leaving many mothers feeling isolated. Another common belief was that newborns born in the seventh month of gestation are more likely to survive than those born in the eighth month. As one mother explained:

“I had such perception, when I mean this, it’s been said that the baby that was born in the eighth month won’t survive, on the seventh they will. So, our mind believes this” (IDI – mother of preterm or LBW newborn).

Mothers and family members reported being unaware of and unprepared for preterm birth and felt lonely, shocked, and confused when the preterm labor started.

“We didn’t go there [to the health facility] to have a baby, we were shocked. We didn’t know what happened, we were confused about what happened, we didn’t go there planning to give birth” (IDI – family member).

A prevailing belief, especially in rural areas, that childbirth is a straightforward process and doesn’t require facility-based care, has contributed to a significant rate of home births. As one participant noted,

“There is a community perception that since mothers have historically given birth at home without issues, there is no need to visit a health facility. This belief is misguided and can lead to complications” (FGD – community members, leaders, and stakeholders).

While caring for preterm and LBW newborns continues to be shaped by deeply held beliefs, there have been improvements in recent years. This progress is largely due to the concerted efforts of the health system at both the facility and community levels, particularly through the involvement of health extension workers (HEWs). Stigma and discrimination by community members toward preterm newborns have declined, and male involvement, especially among fathers, has increased, with more husbands now accompanying their wives to maternal and child health services.

“In this area, the husbands are very willing to help, and they are usually beside mothers to help mothers in providing KMC: so, if mothers are unable or when they sleep, family members provide KMC service” (IDI – NCU healthcare worker).

“I feel the baby improved after I hugged him [held the baby in KMC], and I feel I am contributing to the wellness of my baby. I loved it well” (IDI – father of preterm or LBW newborn).

Despite a significant proportion of mothers, particularly in rural areas, still giving birth at home, there has been a notable shift towards health facility births. The increasing facility birth trend enabled community members to become more familiar with the services provided for the survival and wellbeing of preterm and LBW newborns.

“Nowadays, home deliveries are rare. As a traditional birth attendant, I no longer assist with home births due to the risks, including a lack of vaccinations for newborns. I advise pregnant women to go to health facilities for safe delivery” (FGD – community members, leaders and actors).

***Identification of preterm or LBW newborns***

Participants identified several factors influencing the identification of preterm or LBW newborns during ANC. These included living near a health facility and having access to early ultrasonography. In contrast, those living in remote areas faced limited access to facility-based ANC services due to limited transportation options and inadequate road infrastructure. Other facilitators included access to quality counseling services during ANC and the presence of well-functioning clinics capable of identifying and monitoring high-risk pregnancies. On the flip side, the quality of ANC services was also a concern, particularly at government facilities, due to inadequate staffing, limited availability of essential medications and supplies, and overcrowding. As one mother noted, “They do not check ultrasound regularly because it is a government station” IDI – mother). Weak counseling and information on preterm birth risk factors and mistreatment during visits further contributed to low uptake.

“Health providers didn’t inform me well about the possibility of having a preterm or low birth weight baby they didn’t provide me enough counseling during ANC follow-up” (IDI – mother of preterm newborn).

In areas where HEWs were active, participants reported that they facilitated early identification of preterm births by conducting home visits and educating pregnant women on the importance of giving birth at health facilities and attending regular ANC visits.

Strong family support for pregnant women, such as accompanying them to ANC follow-up visits, and positive family attitudes toward regular antenatal care visits, were also highlighted as facilitators. Conversely, participants identified social and personal factors as barriers to pregnant women’s access to antenatal care. These included delayed recognition of pregnancy, unplanned pregnancies, and a lack of adequate support from family members. In some cases, husbands or relatives actively discourage or restrict visits to health facilities:

“Her husband did not let her go out; he controls her strictly and did not give permission to her for follow-up” (IDI – preterm or LBW newborn grandmother).

Furthermore, many pregnant women were also burdened with household responsibilities throughout pregnancy, leaving them little or no time and energy to prioritize their own health and ANC seeking. In some families, there was a preference for seeking care from traditional healers instead of formal health facilities.

The availability of free ANC services was identified as a facilitator of ANC uptake. However, financial constraints were identified as a significant barrier, as many mothers were unable to afford ultrasound scans, especially when referred to private clinics, or to cover transportation costs to attend regular antenatal care visits – social work support is limited and not universally accessible.

“The other reason that may be a challenge for following ANC is [a lack of money to cover] costs, like transportation costs …” (FGD – general mothers who recently gave birth to term newborn).

Availability of maternity waiting rooms where pregnant women from remote areas stay around their expected date of childbirth, and their labor progression is monitored, was identified as a facilitator for accessing skilled childbirth care services. However, for some mothers, distances to health facilities, transport difficulties, and unreliable ambulance services remain barriers. Even when women reach a facility, challenges persist—such as poor quality of care, inconsistent information about the newborn’s condition, and denial of a labor companion. These experiences contribute to a lack of confidence in the care provided, particularly when families receive mixed messages (e.g., discrepancies between estimated fetal weight during ultrasonography and the actual birth weight) or feel neglected after childbirth.

“… there were a lot of things she needed, but she was told she had finished her treatment, then nobody asked about her, no one checked on her after that; I think there was a little surgery during birth, and she was feeling that pain for a while.” (IDI – family member).

Another key facilitator of the use of skilled childbirth care services was the presence of an adequate number of compassionate, respectful, and caring healthcare workers.

“I don’t know why but they gave me special treatment. Several doctors were here for me … eeeh and, even at birth, there were many, and after birth, they just put her on my chest for a second, they give her oxygen and took her immediately. So, they treated us so well” (IDI – mother of preterm or LBW newborn).

Regarding the availability of essential equipment and supplies, participants recognized adequate equipment preparation for caring for preterm or LBW newborns as an important facilitator. However, stockouts of essential supplies (towels, gloves, IV fluids, etc.) remain a challenge in several facilities.

Consistent with ANC, a growing positive family and community attitudes toward facility births encouraged and supported women during labor and childbirth.

Referral care was facilitated by free of charge ambulance services and, when transportation was not free, some families were able to cover cost while social work support provide financial support to those who could not afford it – “For those mothers who refuse referral out of financial constraints, there are social workers who facilitate to support those mothers financially” (IDI – childbirth healthcare worker). Participants also mentioned a lack of dedicated neonatal ambulances, and those shared with adults are frequently unavailable due to fuel shortages and poor communication between referral liaison officers.

Other facilitators of referral care included the availability of beds at receiving facilities, the use of standardized referral forms to document necessary information, and the presence of healthcare workers to accompany newborns during transfers from the labor wards to NCUs or between facilities. However, referral hospitals often lacked beds due to high caseload. Moreover, ambulances lacked essential equipment such as CPAP and oxygen, compromising the care of sick newborns during transport. Health worker shortages in some of the lower-level facilities also meant they were sometimes unable to accompany newborns during transport.

“…. According to the standard, a neonatal nurse must be present during the referral to take care of the baby. However, in our case, we have only five nurses per shift, and around 34 beds, including the mother’s side. So, five nurses will take care of all 34 beds in every shift ….” (IDI – NCU head nurse).

Mothers’ and families’ understanding and acceptance of the need for referral were critical, as were their preparedness and readiness for referral care, including the ability to cover associated costs. However, some families declined referrals due to cost or beliefs about the newborn’s chances of survival.

***KMC: Skin-to-skin contact***

The successful implementation of KMC was facilitated by adequate infrastructure and resources to support mothers and newborns. These included comfortable and appropriate chairs for KMC practice, KMC binders, adjustable beds, dedicated KMC rooms, and access to private showers and bathrooms.

“There is a binder used to practice a KMC …. We got the binder from the SLL [Saving Little Lives] project. So, we have enough number of these binders that are used to perform KMC” (IDI – NCU nurse).

However, in the majority of the facilities, the lack of adequate infrastructure, including comfortable chairs and beds, which were crucial for mothers, particularly those recovering from cesarean sections was a major challenge. In addition, the KMC wards were often poorly designed, with limited space, failing to provide a comfortable and supportive environment for both mothers and newborns. Furthermore, in some facilities, there was no dedicated KMC room, and essential resources such as KMC binders, preterm-sized diapers, and televisions to demonstrate KMC techniques were absent. The location of the KMC room, which was often far from the labor and childbirth unit, posed additional challenges by delaying timely access for newborns in need of KMC.

“The very small number of beds in KMC and unavailability of space is the biggest gap” (IDI- Birth health workers head).

“While it’s beneficial for them to stay where the baby is, if the patient flow is high, the [KMC] room becomes crowded and suffocated, at times leading to conflicts between the mothers” (IDI – NCU nurse).

Other barriers to the provision of skin-to-skin care included a lack of room-heating systems, the absence of handwashing facilities, and inadequate nearby bathroom facilities, all of which created an unfavorable environment for both mothers and health workers. The overall ambiance of the units in most hospitals was not conducive to the comfort and well-being of mothers and newborns, with unpleasant odors adding to an already suboptimal environment.

Newborns on oxygen therapy faced delays in receiving skin-to-skin contact, as health workers felt they should first be weaned off oxygen. The expression and feeding of breastmilk were also reported to interrupt the continuous provision of KMC. Initiation of skin-to-skin contact was often delayed, particularly for newborns born via cesarean section, as it typically began only after referral to the NCU rather than immediately after birth.

“Since formula milk should be mixed [fortified milk preparation], you have to put them [the newborns] down, so these are the challenges, so hours may be spent on expressing milk and feeding them. It was easy when it [the feeding] was with tubes, but now it might take an hour to feed them a small amount” (IDI – mother of preterm or LBW newborn).

Daily monitoring of KMC practices by health workers, encouragement for family involvement, and provision of detailed guidance on KMC positioning and its benefits through regular counseling sessions and awareness activities supported the adoption of KMC. Success stories shared by health workers helped build mothers’ confidence and motivated them to continue practicing KMC. Educational sessions, starting as early as the newborn’s admission to the NCU and including a structured care schedule and visual aids such as videos and posters in local languages, played a key role in helping mothers practice KMC. However, some facilities lacked a structured schedule to monitor and support consistent practice of skin-to-skin contact.

“They advised me; to check the baby’s breathing, to turn his face to the direction he would look at me, and so that’s how they helped me” (IDI – mother of preterm or LBW newborn).

A positive attitude and recognition of KMC’s importance among health workers inspired confidence and trust in mothers. The presence of dedicated KMC-trained staff facilitated skin-to-skin contact practices. In addition, training played a vital role, with nurses sharing knowledge gained from KMC training sessions with other staff members, even when resources were limited for extensive training.

However, in some facilities, shortages of neonatal health workers, limited training opportunities, and the lack of a designated focal person for KMC contributed to gaps in leadership, coordination, and accountability. Furthermore, some health workers, such as Integrated Emergency Surgical Officers (IESOs) in labor and childbirth units, had minimal involvement in delivering skin-to-skin care.

“It is being practiced in the childbirth room but not in the postnatal ward. We don’t practice it in the postnatal ward, we can’t say we are practicing it when we don’t” (IDI – Childbirth health workers).

“There is a challenge. For example, for the KMC ward, we don’t have a focal person who is assigned for that specifically … So that’s the first challenge I see” (IDI – NCU nurse).

In some cases, participants reported that some health workers paid little attention to KMC, with a few showing negligence and a lack of patience in delivering the care, as reflected by a family member *“* … but some will show negligence, especially at night shift, which means they don’t even check [the baby] once”. A health worker also noted KMC was rarely highlighted as a priority within the childbirth care department.

“They [childbirth healthcare providers] pay less attention to KMC; It is not even raised as an issue in our department. Maybe only in the NCU. We usually transfer the baby to the NCU, and we do not pay attention to KMC [during referral]” (IDI – Childbirth healthcare workers).

A positive attitude and active involvement of mothers and their families were highlighted as key facilitators of continued KMC. Many mothers and family members believed that newborns enjoyed and benefited from skin-to-skin contact; “Yes, liked it [KMC]. Immediately after I put them on skin-to-skin contact, they sleep” (IDI- mother of preterm or LBW newborn). As families observed visible improvements in the newborn’s health, their attitudes often shifted, and they came to value KMC more. Although cultural perceptions around caregiving largely limited fathers’ involvement in KMC, as newborn care was often viewed as a mother’s responsibility. However, some fathers, along with other family members, played an important role in supporting mothers by providing KMC themselves, especially when mothers needed rest or were unable to continue. This supportive care practice not only ensured continuity of KMC but also strengthened family bonds.

“There was a father called [NAME] in our room, and he always hugged the baby. He said, እሷ ብቻለ ምንትደክማለች’ [why should my wife bear the burden alone]” (IDI – mother of preterm or LBW newborn).

Positive perceptions and prior exposure to KMC enhanced its acceptance and implementation. Family members who already held a favorable view of skin-to-skin contact support and participate in its provision – “I had experience with skin-to-skin contact, I helped my wife by giving skin-to-skin contact because she gave birth to twins, and I hugged one baby on my chest to increase the newborns’ temperature. I was happy doing this” (FGD- community members, leaders, and actors). Observing or having prior hands-on experience with KMC enabled both mothers and families to understand its benefits, feel more confident, and be more motivated to adopt the practice. A mother described this “It’s so interesting, hugging her closely feels so interesting; now it feels so wonderful, you see your baby by your side, and it’s interesting” (IDI- mother of preterm or LBW newborn). Keeping mothers who practiced KMC in the same room facilitated peer learning and mutual encouragement, as they shared experiences and techniques.

“… … So, we also create an environment where we share the experience of other mothers. We show them some pictures, and we make a room for mothers to discuss their experience” (IDI – NCU nurse).

Several psychological, cultural, and practical challenges limited the consistent practice of KMC among mothers. Many mothers were reluctant to adopt KMC due to fears of societal judgment or criticism, as they worried about being viewed negatively for using what could be perceived as an unconventional care method. In cases where newborns were unplanned or unintended, families were more likely to resist healthcare workers’ recommendations to practice KMC.

“If babies are born unplanned or unintended, their families are usually against the advice of professionals to perform KMC and other [types of care]” (IDI – NCU health worker).

The physical demands of sitting for long hours and poor sleep while providing skin-to-skin contact led to physical and emotional exhaustion, causing mothers to lose motivation over time, especially during prolonged stays in health facilities. Common fears, such as the newborn falling out of position or concerns over the newborn’s fragility, also created significant apprehension, making it difficult for mothers to fully engage in the practice. Furthermore, some mothers felt embarrassed or skeptical about the benefits of starting KMC immediately after birth. Others experienced feelings of inadequacy or found the practice physically challenging due to discomfort from excessive warmth or the strain it placed on their bodies.

“Sitting for long hours is exhausting, and feeling sleepy, this is common, even now I am doing it alone day and night so there is a problem of not sleeping well, and this exhausted my body, I feel tired” (IDI – mother of preterm or LBW newborn).

Some mothers also felt frustrated when comparing themselves to others, especially when seeing term newborns being breastfed or carried comfortably by their mothers, leading them to abandon KMC after brief attempts. Mothers of twins faced significant challenges in providing KMC on their own and often required family support. However, some family members were fearful of offering skin-to-skin contact due to concerns about harming the small, fragile newborns.

“Since the babies are small, it’s scary, it feels like something would happen to them, on the first day I hugged them I feel like they will vanish inside, I was afraid, actually I don’t carry them lots of days for its so scary, I used to it after I carried them one day; it feels like they are suppressed, I feel like they will stop breathing” (IDI – family member).

The lack of information and awareness about the practice, both before and after birth, was a barrier to providing skin-to-skin contact. Many mothers were not informed about KMC prior to childbirth. Even after birth, counseling on skin-to-skin contact was often limited to the mother, with other family members, such as aunts, not receiving the same guidance, despite their role in supporting the mother and influencing the acceptance of the practice. Additionally, a lack of visual aids in some facilities, such as pictures to guide and remind mothers of the proper KMC techniques, had limited effective practice.

Community awareness about KMC was generally low, with many mothers expressing that little information was shared in their communities. Although HEWs, are primarily responsible for providing education and awareness to pregnant women, KMC was often not discussed or taught during pregnant women forums where HEWs provide group ANC, promote institutional childbirth, and counsel mothers on birth preparedness and complication readiness.

“I doubt that during pregnant mothers’ forum health extension [workers] teach about other issues, not about KMC” (FGD – general mothers who recently gave birth to term a newborn).

“They did not tell me about how long I should give the baby skin-to-skin contact” (IDI – mother of preterm or LBW newborn).

***KMC: Exclusive breastmilk feeding***

Several key factors facilitated the practice of exclusive breastmilk feeding in health facilities. These included implementing tailored feeding plans based on the newborn’s weight, developed in consultation with a neonatologist to ensure compliance with standard protocols; continuously monitoring of vital signs and feeding progress using charts as per the physician’s orders; and creating a comfortable environment for mothers by providing dedicated breastfeeding seating.

“We have a feeding chart. If the nurses are providing the feeding, they sign in the chart every hour they provide” (IDI – NCU head nurse).

Regular health education on key aspects of breastfeeding delivered with clear, positive messages and supported by audiovisual materials played a vital role in promoting exclusive breastfeeding. This impact was even greater when education was extended to the community, to include mothers and influential family members, such as grandmothers, helping to build a strong support network. Additional facilitators included consistent encouragement of mothers to breastfeed exclusively and the use of translators when language barriers arose. Advice on improving milk production, reducing stress, and providing emotional support also helped mothers to sustain breastfeeding.

“They were taught not to give anything until the baby reaches six months … This practice [mixed feeding during the first six months] was common in the past, but now, it has become a culture where no mother gives any food until the baby is six months old” (FGD- community members, leaders, and actors).

However, preterm newborns often faced delays in the initiation of breastfeeding, particularly when they required immediate admission to NCU or lacked the strength to suckle right after birth – “For the first three to four days, the newborns were on oxygen and glucose; they were not having anything, they didn’t have breastmilk” (IDI- mother of preterm or LBW newborn). In cases where mothers were critically ill, anemic, recovering from cesarean sections, or episiotomies, preterm formula was frequently used as an alternative. Formula feeding was also initiated for newborns whose mothers abandoned them or left the facility. Some mothers struggled to express breastmilk in the initial days postpartum. For twin mothers, the challenge of breastfeeding both infants amplified these difficulties.

“She has difficulty because they are twins they both cry at the same time” (IDI – family member).

The presence of compassionate and supportive health workers in some facilities played a vital role in guiding and encouraging mothers throughout the breastfeeding journey. Exclusive breastmilk feeding was achieved when health workers actively monitored postpartum mothers, promptly addressed any breastfeeding challenges, and provided tailored support to those facing specific difficulties, such as unwanted pregnancies, preeclampsia, cesarean deliveries, first-time motherhood, or long birth intervals.

“For mothers who need additional support, especially when the mothers are young and mothers of preterm babies, health workers counsel them about positioning, attachment, and show how to express milk” (IDI – NCU health workers).

Demonstration of proper techniques for breastmilk expression, positioning, and attachment by healthcare workers, while reinforcing the benefits of breastfeeding for both mother and newborn, was a crucial facilitator of breastmilk feeding. Daily updates on newborn’s growth and hands-on guidance with breastfeeding and pump use further motivated mothers. In some facilities, healthcare workers with specialized training in lactation and feeding significantly improved breastfeeding practices by delivering more effective and informed support.

“Especially their kgs [weight] below two or one, and when they are very extremely small, it is mother’s breastmilk, we don’t give other things. ብንሞት እንኳን እንሰጥም [We never give anything other than breastmilk]” (IDI – NCU nurse).

On the contrary, achieving exclusive breastmilk feeding was often hindered by limited support from some healthcare workers. In many cases, assistance was restricted to brief verbal advice, without demonstrations of proper breastfeeding positions or attachment techniques. This lack of hands-on guidance made it particularly difficult for mothers, especially those with small or vulnerable newborns, to maintain effective breastfeeding practices. Without practical support, many struggled to position their newborns correctly and ensure proper latches, which are critical for successful and sustained breastfeeding.

“They [health professionals] didn’t try [to support me feed the preterm baby]; I am the one who is trying. They only told me to feed him” (IDI – mother of preterm or LBW newborn).

“It [the breast] covers their face since they are small you will be challenged to feed them; since she is small, it was difficult to hug her under my breast, she needs to be carried carefully, and such a thing needs patience that was somehow challenging” (IDI – mother of preterm or LBW newborn).

The qualitative study participants also reported that healthcare workers’ lack of training in exclusive breastmilk feeding practices and essential skills, such as KMC, limited their ability to support mothers. Some nurses also faced challenges in assisting mothers with expressing breastmilk, sometimes leading to disagreements. Despite their professional training, some HEWs, influenced by traditional practices, recommend traditional medicines or advise early introduction of foods before the recommended six months.

Regarding breastfeeding resources, several gaps were identified. In the postnatal ward, there was a lack of comfortable chairs for mothers to sit and breastfeed their newborns, and the beds were often uncomfortable due to a shortage of foam mattresses. “There is no chair, normally there is no chair in the postnatal ward. [There is] a bed if she wants to carry her baby and breastfeed, she’s going to sit there, but there is no chair” (IDI – Childbirth healthcare worker head). In addition, breast milk pumps were difficult to obtain in pharmacies. Lack of handwashing facilities and practices, gaps in monitoring exclusive breastfeeding due to the unavailability of feeding monitoring charts, added to the problems.

From a financial perspective, some hospital workers reached out to social workers to help provide a formula for milk fortification, when needed. In some cases, parents donated formula to support other families who could not afford it. Some facilities offered mothers free meals, further supporting their ability to practice exclusive breastfeeding. However, free meals for mothers were not consistently provided in all facilities.

“They are giving us [food] since we came to this room, my families also bring food” (IDI- mother of preterm or LBW newborn).

A positive attitude among mothers and their family members toward exclusive breastfeeding played a crucial role in its success. Most of the families interviewed believed that breast milk was the only suitable nutrition for their newborns. While some traditional practices, such as using traditional medicine like rue when newborns are sick, were introduced by certain family members, several parents do not accept anything unless advised by healthcare workers. “I know that feeding breastmilk is more than anything for the newborns, more than giving them another thing, more than anything, I know mothers’ breastmilk is a hope for their health and survival” (IDI- family member). Despite challenges such as discomfort from a cesarean section wound or episiotomy, mothers continued to breastfeed their newborns. Family support was also vital, as they provide nutritious food to help mothers maintain milk production.

Although there is a widely held positive attitude towards breast milk feeding, attitudinal and cultural beliefs among some mothers and families hinder the practice of exclusive breastmilk feeding. Some mothers, particularly first mothers, opt to feed their newborns formula instead of breastfeeding, believing that their breastmilk is insufficient, especially for preterm or LBW newborns. In some cases, husbands too perceived breastmilk as inadequate, claiming it is too thin and causes frequent vomiting, which discourages mothers further. “They start breastfeeding, but it is not enough. What I mean is the breastmilk is thin, so they frequently vomit” (IDI – family member). Discouragement about breastfeeding in these cases is common, as is the practice of discarding colostrum.

Study participants also reported that some mothers are unwilling to breastfeed female newborns, and traditional practices, such as feeding newborns boiled leaves like “HAMMESA,” and misconceptions that a newborn born preterm cannot survive, contribute to a reluctance to breastfeed. The changing urban lifestyle led some mothers to stop breastfeeding within two or three months.

“… in this society, it is encouraged to give birth to a male baby, so sometimes we face mothers who do not want to breastfeed since they have a female baby” (IDI – Childbirth healthcare worker head).

There are skill gaps among mothers, particularly primiparous mothers, in appropriately holding the newborn for feeding. In some mothers, inverted nipples affect breastmilk feeding. Furthermore, some mothers may not accept the counseling offered, because they feel dissatisfied or unsupported, particularly when material needs, such as newborns’ clothes, are not met.

“Most of the primies [first-time mothers] don’t have the skill to hug babies. Even the multis can’t; from our observation, they may be confused” (IDI – NCU nurse).

***Discharge and follow-up care***

Our study identified several enablers that contribute to successful discharge and follow up care of preterm newborns. These include the use of written discharge criteria; having a dedicated counselling corner and provision of quality counselling for mothers and family members; availability of dedicated physician who evaluates the newborn before discharge, including checking the newborn’s breastfeeding ability, vital signs, and overall stability; and conducting lab investigations and weighing the newborns to assess progress. Newborns are typically transferred from the NCU to KMC ward rather than directly to their homes, which ensures a smoother transition and continued support. Participants also reported absence or frequent interruption of lab investigations.

Conversely, a lack of focus on discharge counseling among healthcare workers in some facilities was identified as an important barrier. Many healthcare workers feel their attention is more focused on other tasks and place insufficient emphasis on ensuring that mothers and families are adequately prepared to care for their newborns at home. Even when they have time for such discussions, some health professionals often do not provide the necessary guidance on post-discharge care. Additionally, for clients coming from remote rural areas, a language barrier further complicates communication and the understanding of essential care instructions.

“… it [discharge counseling] is something we should strengthen but, as I said, we do not have enough number of [healthcare] workers. For example, you saw the nurse asking for the discharge paperwork, but immediately a new case arrived, and she ran there [to manage the new admission]. I am not sure if she manages to counsel the discharged patient …” (IDI – NCU nurse).

Our analysis revealed that, in some facilities, healthcare workers maintain contact with mothers through phone calls and scheduled follow-up visits. They offer guidance and ensure the newborn’s health is stable before discharge, while also actively encouraging fathers to participate in KMC and provide ongoing support to mothers at home. To facilitate continued assistance, families are provided with contact information, such as phone numbers, so they can reach out whenever they need help. For families in remote areas, contact details are shared to enable communication with HEWs. During home follow-ups, when active, HEWs monitor newborn care. Although not consistently practiced, some healthcare workers reported taking the personal initiative to contact the HEWs, who are familiar with the families, and request them to monitor the status of preterm or LBW newborns within the community.

“There is a community KMC, we follow them through phone calls, also we check them during their return visit by appointment” (IDI – NCU nurse).

Barriers to effective discharge and preparation of women and families for home care are highlighted. These included distance of facilities from mothers’ residence; for some mothers, absence of a strong mechanism to monitor newborns at home after discharge [“After they go home, we don’t have mechanism to check” (IDI – NCU nurse)], lack of provision of food and support during their stay in some of the facilities, including the lack of sanitation materials and diapers [“Our main challenge is unavailability of support for mothers whose newborns are admitted such as food, sanitation materials, diapers and others … patients leave for home against the doctor’s advice” (IDI – NCU nurse)]. Mothers who have other children at home found it difficult to stay long in the facility and often requested early discharge.

Financially, some facilities waive service fees for families if cost is identified as the reason they leave against medical advice. Additionally, families are sometimes provided with essential supplies, such as diapers and baby clothes, to ease the transition to home care. This not only solves the immediate problems faced by families but also fosters positive community perceptions towards hospital-based care.

However, financial constraints remain a significant barrier to ensuring proper care at home. Many families struggle to afford necessities such as water, soap, diapers, and food, which are crucial for both the mother’s and the newborn’s well-being. Limited access to recommended foods and the lack of post-discharge support often leave mothers feeling exhausted and overwhelmed. These financial difficulties also affect follow-up visits, as mothers may not have the means to travel back for post-discharge checkups.

“Financially, we are not capable of buying the necessary things for the newborns. It is difficult to prepare everything” (IDI – family member).

Some mothers and families felt confident and prepared to care for their newborns at home after discharge. This confidence stems from the newborn’s improved health status at the time of discharge and the guidance provided by healthcare professionals. Most families and mothers accept the decisions and advice of health professionals regarding continued care at home. In many cases, mothers receive additional support from family members and neighbors.

“Yes, they [mothers] are confident, they are confident because experts or the health professionals make a decision. In the past, mothers were taught by their own mothers how to handle their babies. But this time, they give birth at the hospital. Here, the professionals advise and send them home after confirming the overall health of the baby, so they are confident about their preterm or LBW newborns” (FGD – community members, leaders, and actors).

However, some mothers expressed doubt about providing care after discharge due to the absence of support at home. Some mothers also felt they did not have enough time to care for the newborn properly due to household responsibilities. Some parents and family members are reluctant to follow professional advice, especially when the newborn is unplanned or unwanted.

“As I told you previously, my problem is the interruption and challenge of not getting help. As I told you previously, after coming here, no one is at home to support me, so I could only hold the baby at night. I will hold him on my chest until I fall asleep …” (IDI – mother of preterm or LBW newborn).

***Introduction of iKMC***

Mothers were not yet supported to provide iKMC at the time of this study; we therefore asked participants about their attitudes towards introducing iKMC in the facilities and the adoption of this care by mothers and families. Facility managers were optimistic and expressed their commitment to the successful implementation of iKMC by leveraging proven collaborative teamwork within the NCU. iKMC was considered relatively feasible to implement alongside other neonatal care practices, particularly when supported by a well-equipped and efficiently functioning NCU. Despite challenges, hospital managers expressed a commitment to implementing iKMC because they believed it offered substantial benefits to newborns.

“The collaborative model we use in the NCU will serve as a blueprint for the iKMC approach. By applying the same strategy—integrating midwives, pediatricians, nurses, and oxygen professional, we can ensure a seamless and effective service delivery” (IDI – Health facility manager).

Likewise, healthcare workers held a positive attitude towards and were supportive of iKMC. They perceived iKMC as feasible to implement and reiterated that its unique advantage lies in fostering close physical and emotional bonds between mothers and their newborns. They reported that iKMC could be effectively provided if there were collaboration between maternity and NCU staff. With committed personnel available and the belief that iKMC does not require advanced technology, they viewed it as a relatively straightforward and cost-effective approach. Furthermore, they felt that iKMC is aligned with the health facilities’ overarching goals of reducing neonatal mortality and enhancing the quality of maternal and neonatal care.

“The main requirement for implementation is space; we do not need new scientific advancements or sophisticated technology. Therefore, I believe that providing this immediate care should not be particularly challenging” (IDI – Health facility manager).

“The other thing, relatively it [iKMC] seems cost-effective, it is viewed as enhancing the care without investing in any special thing. You don’t use a special resource to implement iKMC, but you improve the outcome” (IDI – Health facility manager).

Despite positive initial responses to adopting iKMC, some participants also identified a potential barrier related to healthcare workers’ attitudes, as they were familiar to caring for newborns in the NCU without their mothers present, only involving mothers once the newborns were stable.

“The other is the attitude of the healthcare providers; we healthcare personnel usually are familiar with keeping the baby in ICU until the baby is stable, so that we can bring him to mother’s side. So, asking health professionals who are used to this trend to do iKMC at NCU will be challenging” (IDI – Health facility managers).

Some felt that implementing iKMC would be challenging in their current work environment, especially given the shortage of healthcare workers and the high staff turnover, which made it difficult to maintain a skilled and experienced workforce. Some participants felt that the heavy workload would limit the healthcare workers’ ability to adopt new approaches. In addition, as iKMC would be a relatively new initiative, many healthcare workers had not received adequate training in its implementation.

“… However, the common problem here is finding well-trained professionals. It’s not that training isn’t available, but for example, after completing NCU training, many professionals work for just two months before moving to another facility” (IDI – NCU nurse).

Mothers and families generally showed a positive attitude and strong will to embrace iKMC, as they felt it would significantly benefit newborns and help build a strong bond between mother and newborn. Mothers appreciated the importance of being with their newborn from the start with a belief that iKMC would provide essential warmth and comfort. They felt it would create an irreplaceable connection and enhance the newborn’s chances of survival. While some mothers recognized potential challenges, they expressed a readiness to implement iKMC without hesitation if it benefits their newborn’s health. Many emphasized that the care would not be difficult, especially compared to the effort of carrying a fetus during pregnancy.

“As long as it is useful for the baby and the warmth he would get from the mother is enough and helpful to keep him alive, I think it’s possible” (IDI – mother of preterm or LBW newborn).

“Mother wouldn’t hesitate to sacrifice anything, she will say ok, she will agree strongly because while they were a part, she was suffering longing for them very much, she will be happy to stay by their side, that is a great joy for a mother, whatever it is sleeping hugging a baby is a great thing in my opinion” (IDI – family member).

On the other hand, the thought of practicing iKMC while the newborn is on a CPAP machine, made some mothers feel particularly overwhelmed. Others felt that their own health challenges, such as fatigue or postnatal recovery, may make it hard for them to sustain the practice. Some also worried that family members or visitors might perceive them or their newborn as unwell or inadequately cared for. In the absence of prior examples or success stories, some mothers were unsure about the effectiveness of iKMC. Some families feared disapproval from some community members and elder relatives might further discourage its acceptance.

“It is difficult for me to do it because your baby is on a machine and holding him like that is difficult, it is very frustrating. The food tube [nasogastric tube] is normal but the oxygen and especially the machine that supports their hearts in NCU [CPAP], the sound is disturbing. So, as a mother staying there hearing all that is difficult, it is difficult for me, and I don’t think I would do it” (IDI – mother of preterm or LBW newborn).

The community stakeholders were also receptive to iKMC and view it as having a transformative impact on neonatal health, particularly for preterm newborns. Younger women were especially open to adopting the practice if provided with proper counseling. There was a shared belief that iKMC realizes the mother’s unique role in providing life and comfort. Many noted that no one in the community would disapprove of iKMC, given its clear benefits for neonatal survival. Mothers found reassurance in the practice, often saying that just as the fetus was once in the womb, the newborn is now between the breasts, making the transition natural and manageable.

Although mothers and families generally expressed willingness to embrace iKMC, some mothers expressed concerns and hesitation about providing it. A few found the idea frightening, describing it as difficult or unnatural to hold their newborn against their chest, especially when the newborn is small and fragile. While watching the iKMC video, one of the mothers said it was both very emotional and frightening. She said, “It is difficult, I do not know [she started crying]” (FGD – recent mothers). Healthcare workers and community members also expressed concerns that providing iKMC could restrict movement, disrupt sleep, and cause fatigue from carrying the newborn.

“It’s not just related to fear, but personally, I have concerns that it could make things harder for the mother. For example, when the baby is carried on her [the mother’s] chest, whether sitting or lying, it limits her ability to move freely. It might be uncomfortable for her to sleep on her side, and she may need to lie on her back, which could be uncomfortable. The extended time spent carrying the baby could also cause fatigue” (FGD – community members, leaders, and actors).

***Creation of M-NCU***

To effectively establish the M-NCU, the availability of several key infrastructural inputs was seen to be crucial. In some facilities, adequate space, essential facilities such as showers, toilets, and sanitary materials for mothers,and customized, comfortable beds specifically designed for iKMC were perceived to significantly enhance mothers’ comfort and willingness to participate in iKMC. Efforts to repurpose and renovate rooms essential for iKMC were already underway, demonstrating a strong commitment to this initiative. Upgrading the NCU to have a continuous water supply and ensuring a well-structured, hygienic environment is recognized as an enabler of successful M-NCU care provision.

“We are also exploring solutions to address the space issue. We are renovating rooms adjacent to the childbirth area. One of these rooms will be repurposed for iKMC, which will help address the distance issue between the NCU and OBGYN [obstetrics and gynecology] departments” (IDI – Health facility manager).

On the other hand, participants highlighted that, in some facilities, infrastructural challenges were perceived as significantly hindering the implementation of M-NCU. The considerable distance between labor and childbirth wards and the NCU complicates the integration of iKMC practices. In addition, lack of suitable or adequate spaces presents a major barrier, as current setups often fail to support M-NCU care. A shortage of beds further exacerbates the issue, preventing zero separation between mothers and their preterm or LBW newborns.

Even in functioning NCUs, the environment is often overcrowded and stressful in some facilities. Basic infrastructure, such as proper functional handwashing facilities, is often lacking, while electricity-related challenges, including the absence of a continuous power supply, particularly in birthing facilities, create further obstacles. Participants emphasized the urgent need for investment and redesign to ensure the successful implementation of M-NCUs.

“The other thing is, there is no infrastructure like the one we saw in the video; my neighbor’s wife was sent home because there is a shortage of beds in the hospital. Therefore, from both sides, which means from the family and the health facility, [there are] problems that I raised here. It is not easy” (FGD – community members, leaders, and actors).

From a supply and equipment perspective, participants felt that the availability of essential tools in the existing NCU serves as a significant foundation for creating a well-equipped M-NCU. Functional CPAP machines, digital weighing scales, and the availability of oxygen, necessary medications, radiant warmers, nasal prongs, and similar equipment are crucial components that ensure quality care for newborns. In the faith-based hospital, identified as iKMC-eligible facility that serves its catchment populations free of charge, continuous donor support plays a vital role in procuring supplies such as jaundice meters, which are otherwise difficult to source locally. These resources collectively enhance the M-NCU’s capacity to deliver comprehensive and effective care, meeting the needs of both mothers and newborns.

On the contrary, some facilities reported that widespread shortages of essential equipment and supplies in existing NCUs would pose significant challenges to the implementation of M-NCU. Critical items such as oxygen, properly sized nasal prongs, CPAP machines, suction machines, radiant warmers, phototherapy machines, thermometers, and monitoring devices are often in short supply. Frequent shortages of medicines, gloves, distilled water, and other essential materials exacerbate the situation, while infection prevention and control (IPC) supplies, including gloves, soap, towels, alcohol, and gowns for parents, are often insufficient. Staff face similar shortages of necessary attire, such as shoes and clothing, required for entry into the NCU.

“We do not have enough CPAP; there are no prongs, and oxygen shortage. We do not have newborn size prongs, and we use larger size which causes a wound in the baby’s nose” (IDI – NCU head nurse).

According to health facility managers, establishing of an M-NCU is both feasible and beneficial, particularly if supported by government budgets and contributions from various partners, as the associated costs can be effectively managed. In fact, participants believe that M-NCU implementation has the potential to reduce overall expenditures by minimizing mother-newborn dyads’ hospital stays and promoting faster recovery through infection prevention.

“Practically, iKMC can result in faster recovery for babies and fewer infections, which shorten hospital stays for mothers and thus reduce overall costs” (IDI – Health facility manager).

Conversely, participants highlighted that establishing M-NCUs faces significant challenges due to the reliance on donations as the primary source of materials needed to support small and sick newborns. This dependency raises concerns about the long-term sustainability of care, as continuous support is necessary to maintain operations. The absence of a specific financing mechanism for such initiatives within the health system limits its widespread and sustainable implementation. They also thought that allocating government funds for iKMC services can be a lengthy process, if even possible, and financial barriers are likely to remain the biggest challenge in implementing and maintaining M-NCU care. Participants believed that, considering this, it is practical to begin on a small scale, utilizing existing resources and addressing challenges as they arise.

“You know including radiant warmer materials for newborn care are costly and not easily available on the market hence we cover that majorly through donation. If there is no donation even if it is possible to begin the [iKMC] implementation, we may be challenged to ensure the continuity” (IDI – Health facility manager).

## Discussion

This study aimed to assess the health system’s readiness for integrate iKMC for preterm and LBW newborns into Level 2 SSNC facilities in Ethiopia using a mixed-methods approach. Despite several challenges, the health system, families, and communities were receptive to the integrating iKMC into inpatient NCUs, and the health system exhibited some level of readiness to enable this.

### Identification of preterm or LBW newborns

Quality antenatal, childbirth, and referral care provision is key for identification of pregnant women with high risk of preterm or LBW births or iKMC-eligible newborns. The facility mapping data showed that all four iKMC-eligible facilities offered routine antenatal ultrasounds, ANC services with protocols for identifying high-risk women, and antenatal breastfeeding counseling. However, the qualitative findings highlighted shortages of essential equipment to provide the services. This discrepancy might be explained by service interruptions, occasional device malfunctions, or bias in facility mapping data due to positive self-evaluation of service availability. On the other hand, most facilities lacked antenatal KMC counseling, which could hinder parents’ preparedness to embrace and implement iKMC right after birth, as recommended by WHO. ^24^

While the availability of quality ANC in some facilities improved uptake, poor-quality ANC was reported in others. Mistreatment during ANC visits and overcrowding at these facilities compromised service quality and limited care availability during follow-ups. Other researchers have previously identified these persistent health system challenges ^25^^,^^26^ and which need to be addressed, as poor quality ANC is associated with risk of having preterm or LBW newborns. ^27^^,^^28^

The majority of births, and most of the preterm or LBW births, occur in CEmONC facilities with inpatient NCUs. In addition, despite the extensive network of facilities providing childbirth care services in the study area, most newborns eligible for iKMC who presented to the BEmONC facilities were in-utero referred. This makes it feasible to integrate iKMC within the Level 2 SSNC units of select facilities, in alignment with WHO recommendations. ^29^

Previous studies have documented suboptimal childbirth care standards and negative family experiences. ^30^^,^^31^ Our findings were mixed. The facility mapping found that all iKMC-eligible facilities permitted a companion during labor and birth, administered antenatal corticosteroids, and had trained health workers to care for preterm or LBW newborns. In addition, although insufficient, all four facilities were equipped with functional resuscitation corners, radiant warmers, preterm-sized equipment, digital weighing scales, and handwashing facilities. While maternity waiting rooms and the availability of an adequate number of compassionate, respectful, and caring healthcare workers in some facilities were seen as facilitators, qualitative study participants reported significant gaps in the quality of childbirth care and a lack of users’ trust in the services provided. Although facilities reported allowing birth companions, some families experienced the denial of labor companions (family members) during childbirth. As having a female birth companion (surrogate mother) is key to iKMC initiation in the labor room or OTs, the denial of a birth companion might limit families’ willingness and ability to initiate iKMC. Quality childbirth care is critical for iKMC, and its standardization requires urgent attention. Strengthening this area requires targeted interventions that build on existing facilitators and address identified gaps to ensure consistent and effective implementation.

Unavailability of ambulances, uncooperative ambulance drivers, de-prioritization of preterm or LBW newborns in ambulance service allocation, and rejection of referrals by receiving facilities have been documented as barriers to referral care in previous studies. ^32^^–^^35^ Similarly, our study identified several challenges that add nuances to the factors limiting referral care for preterm or LBW newborns. The lack of dedicated neonatal ambulances and frequent fuel shortages hinder timely transfers. While all facilities reported communicating with the receiving facility through a liaison office before referral, they also noted a lack of prior communication when receiving newborns referred from lower-level facilities. A similar practice was reported in a study from Nigeria, in which 78.6% (22 out 28) of facilities assessed rarely or never contacted the receiving facilities prior to referral. ^36^ This highlights a gap between liaison officers, who facilitate referrals, and the staff who receive the newborns referred.

In addition, limited newborn unit capacity, staffing shortages to provide care at the receiving end or accompany during transfer, a lack of essential ambulance equipment (CPAP, oxygen), and reliance on public transport for referral compromise care during transport. Consistent with this, in a study conducted in Ibadan, Nigeria, only 4% of newborns arrived at the receiving facility by ambulance, 7% were accompanied by medical personnel, and 3.5–3.7% had Ambu bags, oxygen, and some drugs available during their transport. ^37^ Furthermore, our study found that some families decline referrals due to financial barriers and concerns over survival rates. For preterm or LBW newborns delivered at lower-level facilities or home, addressing these barriers is critical to ensuring that these vulnerable newborns reach an iKMC-eligible facility in time to benefit from iKMC.

### KMC: Skin-to-skin contact

The facility mapping data showed that all four iKMC eligible facilities provide KMC services for preterm or LBW newborns. Previous studies reported facility leadership support, availability of space and supplies, KMC policies and protocols, supportive staff policies, and supportive supervision as key facilitators of KMC provision. ^38^ Similarly, facilitators of skin-to-skin contact identified by our study include availability of dedicated rooms/spots, shower or bathroom, supplies such as KMC chairs and binders, training opportunity for staff, supportive attitude of staff and family members towards KMC, use of visual aid, and monitoring, and encouraging and providing detailed guidance to mothers and family members. The qualitative data also identified the absence of these facilitators as barriers to the provision of KMC. In addition, provision of KMC was constrained by factors including distance between childbirth and neonatal ward, shortage of healthcare workers for neonatal care, psychological (criticism from communities on adoption of unconventional care), cultural (limited involvement of fathers in neonatal care) and practical (availability of resources, sitting for long hours, having twin births, cesarean childbirth, newborns still on CPAP or oxygen) challenges mothers face to practice KMC. With strong supportive leadership, these challenges are surmountable, ^38^ and overcoming them to ensure provision of quality KMC for stable newborns is critical to creating an enabling facility environment for introducing iKMC for unstable preterm newborns. Previous implementation research has highlighted several key strategies for effective KMC provision, which can be replicated to enhance KMC practices. These include dedicated KMC champion healthcare workers, empowerment of mothers, continuous improvement through data-driven models, and enhanced connections between the community and health systems. ^39^

### KMC: Exclusive breastmilk feeding

In addition to initiating immediate skin-to-skin care and providing it for 8–24 hours per day, another critical component of iKMC is feeding preterm or LBW newborns with breastmilk. WHO’s feeding recommendations for LBW newborns include early initiation of breastfeeding when possible, providing breastmilk until direct breastfeeding can begin, exclusive breastfeeding for the first six months, and continued breastfeeding up to two years of age. ^40^ The facility mapping revealed that all four iKMC-eligible facilities initiated breastfeeding within one hour of birth for stable newborns. They also provided assisted feeding, intravenous fluids, and medication to small and sick newborns. Previous studies highlighted duration of hospital stay, early initiation of breastmilk expression, access to practical professional support, positive breastfeeding sentiment, social support, breastfeeding control, knowledge about breastfeeding, and intentions to breastfeed as facilitators. ^41^^,^^42^ Our qualitative data identified a similar set of facilitators of exclusive breastmilk feeding, including positive attitudes towards breastfeeding shared by families and healthcare workers, personalized support for mothers, demonstrations of breastmilk expression and breastfeeding techniques, and social support. An additional facilitator identified in our study is the financial support provided to mothers unable to afford fortifier formula, which is critical for feeding preterm newborns.

However, as widely documented in the literature, barriers to exclusive breastmilk feeding remain significant. ^43^^–^^45^ The barriers our study identified included inadequate support, limited access to resources such as breast pumps, the absence of comfortable seating and handwashing facilities, and a lack of food provision for mothers by hospitals. Additional challenges arise from maternal or neonatal illness, twin births, and cultural attitudes and practices, such as discarding colostrum, beliefs that breastmilk is insufficient or inferior, reluctance to breastfeed female newborns, and the traditional practice of feeding newborns boiled leaves. As early breastmilk feeding is an integral part of iKMC, effective health system strategies are needed to address these challenges.

### Discharge and follow-up care

The fact that preterm or LBW newborns are discharged alive from the hospital does not guarantee their safety after leaving the facility. A significant proportion, particularly those weighing less than 1,200 grams, die post-facility discharge. ^46^^,^^47^ The WHO advises that small and sick newborns should be discharged from the hospital only when home care is deemed safe, and caregivers have received a discharge management plan and demonstrated competence in newborn care. ^48^ However, in practice, a significant number of mothers, even when feeling prepared for discharge, often return for unplanned post-discharge care. ^49^ Our qualitative data identified several factors that both support and hinder effective discharge care practice.

The use of standardized discharge criteria, the presence of dedicated physicians for pre-discharge evaluation, and comprehensive counseling for mothers and families contributed to a smoother transition from hospital to home. In addition, waiving service fees for financially struggling families and providing essential supplies, such as diapers and newborn clothes, helped extend the length of stay of families who would otherwise leave against medical advice and improve their discharge readiness. Similar interventions at St. John’s Hospital in India reduced the rate of leaving against medical advice. ^50^ The newborn’s health status at discharge, along with healthcare workers’ guidance, helped build caregivers’ confidence. Maintaining post-discharge communication through phone calls and scheduled follow-up visits further strengthened continuity of care.

Conversely, the absence of these enabling factors presents significant barriers to effective discharge and follow-up. Furthermore, lack of support at home, competing household responsibilities, long distances to healthcare facilities, and weak connections between hospitals and HEWs hindered proper follow-up care. Cultural and traditional home care practices, such as the giving of newborns herbal medicines like “ *Hamessa*” pose additional risks to newborns. Moreover, when a newborn is unplanned or unwanted, parental motivation to follow healthcare workers’ advice and adhere to follow-up care could be diminished. Previous studies have found that limited parental involvement and partnership in a newborn’s care, inadequate information provided to parents, community stigma surrounding preterm births, parents’ perceptions of the family-centeredness of care, low parental self-efficacy, and high parental anxiety all affect parents’ preparedness for discharge and their ability to provide post-discharge care for small and sick newborns. ^51^^–^^53^ Expanding the facilitators and overcoming the related barriers to effective discharge and follow-up care is essential to ensure that newborns receive the full benefits of iKMC and are protected against the risk of death.

### Introduction of iKMC

Overall, despite existing and anticipated challenges, the introduction of iKMC for unstable preterm newborns was regarded as beneficial and feasible by facility managers and healthcare workers. They emphasized the importance of fostering strong collaboration between maternity and neonatal teams and integrating this model into existing neonatal care practices. Although some mothers, families, and communities expressed apprehension about providing iKMC to unstable newborns receiving intensive care, most expressed a positive attitude and a strong willingness to adopt iKMC, recognizing its benefits for newborns and its role in fostering an irreplaceable bond between mothers and their newborns. This predominantly receptive attitude towards iKMC among facility managers, healthcare workers, mothers, and families creates a strong foundation for integrating the intervention into the SSNC.

### Creation of M-NCU

Successful iKMC implementation requires transforming and standardizing the NCUs into M-NCU, ^14^ allowing mothers or surrogates to stay with their preterm newborns 24/7 to provide continuous skin-to-skin contact and additional care based on their needs. iKMC also necessitates integrating maternity and neonatal care teams to initiate care immediately after birth and continue it within the M-NCU. The iKMC-eligible facilities are staffed with obstetricians, neonatologists or pediatricians, NCU nurses, and midwives. A significant proportion of these healthcare workers have received training in managing preterm birth and the deliverying SSNC, creating a strong foundation for integrating iKMC into the existing care system.

Implementation of effective iKMC also requires strengthening the SSNC quality, as initiating skin-to-skin contact immediately after birth alone cannot significantly improve neonatal outcomes. It should be implemented as an integral component of the SSNC. Studies documented the limited readiness of health facilities in Ethiopia to provide inpatient care for small and sick newborns. ^33^^,^^54^^,^^55^ Our facility mapping data revealed that all four iKMC-eligible facilities have some equipment for thermal care, including room heaters, radiant warmers, and incubators, although these resources are insufficient. CPAP is the primary mode of respiratory support, but two facilities use home-grown CPAP systems with 100% oxygen with the risk of retinopathy of prematurity. ^56^ Infection prevention practices varied, with all facilities reporting handwashing stations at the entrances of their newborn units. However, water supply interruptions were a recurrent challenge in three of the facilities. While hand rubs and alcohol were used inside the units, proper handwashing compliance was not observed in three facilities, showing the gaps in infection prevention practices.

Similarly, our qualitative data showed infrastructural challenges, including the unavailability of adequate space, distance between birth and neonatal wards, shortage of beds, handwashing facilities, toilets, and showers for mothers, which would be the main barrier to implementation of M-NCU, and design innovations will be required to transform the existing NCU to M-NCU within the available space constraints. Furthermore, shortages of essential equipment and supplies in existing NCUs present a significant challenge. Frequent shortages of medicines, distilled water, and other essential materials exacerbate the situation. At the same time, infection prevention and control supplies, including gloves, soap, towels, alcohol, and gowns and shoes for parents, are often insufficient. The financial investment required to address these gaps is unavailable because the health system lacks earmarked investment for SSNC and neonatal health more broadly. ^33^ Facilities, regional health bureaus, and the Ministry of Health need to invest in SSNC to address these gaps and collaborate with the SSNC partners to mobilize resources, ensuring that facilities are adequately equipped to integrate iKMC into functional SSNC units.

### Strengths and limitations

The current study has several strengths. We used a mixed-method approach that integrated quantitative facility mapping and qualitative assessment, providing insights from multiple perspectives enabling a comprehensive evaluation of the health system’s current readiness to introduce iKMC. To enhance the credibility and trustworthiness of the findings from IDIs and FGDs, several measures were applied. We recruited a diverse group of research assistants, including individuals with backgrounds in public health and sociology, young and older, male and female, and from the Sidama region and Addis Ababa, to ensure that interviews were approached from multiple perspectives. In addition, to avoid missing pertinent findings and capture inputs from participants who were native speakers of local languages, interviews were conducted not only in Amharic but also in the Sidama Afoo language. To diversify context, participants were recruited from urban, semi-urban, and rural communities. Ongoing supervision was conducted, including debriefing sessions throughout the data collection process, both in person and via virtual meetings, to improve data collection process based on lessons learned. An interim analysis workshop was also held to provide an overview of the findings and guide the focus of the subsequent data collection. Finally, to validate and disseminate the findings, a workshop was held, attended by higher officials, representatives from the regional health bureau, various stakeholders, health facility managers, and healthcare workers from the study area, who provided suggestions and comments that strengthen the final write-up.

The study also had limitations. First, the study site was selected based on criteria including strong leadership commitment and the availability of basic childbirth and SSNC services, which have been strengthened through the Saving Little Lives program. Although these criteria were essential for introducing the iKMC program, the findings from the facility mapping and qualitative assessment might not reflect realities in contexts where these conditions are absent. Second, while all BEmONC and CEmONC facilities within the iKMC study area were included, the data reflect the health system and socio-cultural context specific to that region, limiting the generalizability of the findings to subnational or national levels. Nevertheless, we believe the insights documented in our study are transferable to other locations within Ethiopia or beyond that share similar health systems or socio-cultural contexts. Third, during the analysis and manuscript preparation, iKMC was introduced in three of the four eligible facilities. Throughout this phase, co-authors actively participated in various virtual and in-person discussions, training sessions, mentorship activities, and co-design workshops. While every effort was made to maintain neutrality in analyzing and interpreting the pre-implementation data, it is important to acknowledge that our engagement in the iKMC implementation may have influenced its interpretation to some extent. Finally, although facility mapping data collectors received training and supervision to enhance the validity of the quantitative data they collected, health workers, service unit leaders, and health facility managers may still tend to assess the quality of care and services they provide favorably. ^57^

## Conclusion

Despite significant health system gaps that have limited the provision of standard care, hospitals with inpatient NCUs attend the majority of births, including most preterm and LBW newborns. While resources remain insufficient, some of these hospitals do have essential equipment, supplies, staff, and infrastructure to identify and care for preterm and LBW newborns. Importantly, they have shown openness to adopting the iKMC approach and establishing the M-NCU model of care. To effectively integrate iKMC into Level 2 SSNC units, we recommend that the Ministry of Health, regional health bureaus, and SSNC implementing partners build on these existing facilitators and address the barriers to iKMC implementation identified in this study by designing targeted health system strategies.

## Consent

Written informed consent for publication of de-identified formative assessment and qualitative data was obtained from the study participants.

## Author contributions

ASE, SR, SY, and AP conceptualized the study, designed the methodology, and supervised data collection and analysis. FWB, AA, MSA, and MS contributed to data collection, analysis, and interpretation. HN, SG, and DWK synthesized the qualitative data and drafted the initial qualitative results. AA analyzed the facility mapping data and prepared the preliminary quantitative findings. ASE drafted the first version of the manuscript. ASE, HN, SG, DWK, AA, MSA, MS, FWB, SR, SY, and AP critically reviewed the draft and enhanced its intellectual quality. All authors reviewed and approved the final manuscript for publication.

## Data availability

The data for this study consist of quantitative formative assessment data and qualitative IDI and FGD data. Because consent was not taken from participants to openly publish either of the data on a repository data access will be granted up on request by users and on explicit data sharing agreement that safeguards the confidentiality of facilities, healthcare workers, and families and provides intended plans for accessing the data Requests for access to the data may be submitted via email to the principal investigator at abiy.seifu@aau.edu.et.

### Reporting guidelines

COREQ and SRQR forms.
